# Complete Mitogenome of *Chimarrogale styani*, Phylogenetic Placement Within Soricinae, and Exploratory Mitogenomic Comparisons

**DOI:** 10.3390/genes17101244

**Published:** 2026-10-09

**Authors:** Xiujia Wu, Tao Wen, Guangzhi Chen, Wenge Dong

**Affiliations:** Key Laboratory of Ectoparasite Systematics and Evolution of Yunnan Provincial Education Department, Institute of Pathogens and Vectors, Dali University, Dali 671000, China; wuxiujia1025@163.com (X.W.); 14727676620@163.com (T.W.); yanmiechenyuan@163.com (G.C.)

**Keywords:** Soricinae, mitogenome, phylogeny, codon usage, lifestyle

## Abstract

**Background/Objectives:** Semi-aquatic lifestyles may impose distinct energetic and physiological demands, but their association with mitochondrial genome evolution remains poorly understood in soricines. This study aimed to characterise the complete mitochondrial genome of the Chinese water shrew, *Chimarrogale styani*, clarify its mitochondrial phylogenetic placement within Soricinae, and evaluate whether mitochondrial genomic characteristics differ between semi-aquatic and terrestrial soricines after accounting for phylogenetic non-independence. **Methods:** An adult *C. styani* specimen collected from Hongpo Village, Deqin County, Yunnan Province, China, was used for mitochondrial genome sequencing. The complete mitogenome was assembled and annotated; Soricinae relationships were reconstructed using 13 protein-coding genes and two rRNA genes, and mitochondrial composition, codon usage, amino-acid properties, and Ka/Ks ratios were compared between selected semi-aquatic and terrestrial soricines. Phylogenetic generalized least-squares analyses were used to account for phylogenetic non-independence. **Results:** The *C. styani* mitogenome is 17,207 bp long and contains the typical vertebrate mitochondrial gene set. Phylogenetic analyses supported Soricinae monophyly and placed *Chimarrogale* as sister to *Nectogale*, with *C. styani* clustering with *C. leander*. Semi-aquatic taxa had higher AT content and T3s values, whereas terrestrial taxa showed stronger bias toward preferred synonymous codons. Semi-aquatic taxa also showed slightly higher average protein hydrophobicity. Most compositional and codon-usage differences remained significant after phylogenetic correction. Evolutionary-rate differences were gene-specific: *COX1* and *ND6* showed higher Ka/Ks ratios, whereas *ND3* and *ND4L* showed lower ratios in semi-aquatic taxa; all mitochondrial protein-coding genes remained under purifying selection. **Conclusions:** These results provide a genomic resource for *C. styani* and support lifestyle-associated mitochondrial variation in soricines, while indicating heterogeneous gene-specific evolutionary responses. Broader taxon sampling, additional independent ecological transitions, and nuclear genomic data are needed to further evaluate the relationship between lifestyle and mitochondrial genome evolution.

## 1. Introduction

The Soricinae comprises a highly diverse group of shrews, exhibiting a wide range of ecological lifestyles, including terrestrial, semi-aquatic, semi-arboreal, and semi-fossorial forms [1,2,3]. These distinct lifestyles are associated with pronounced morphological and molecular divergence among lineages. Despite substantial progress in resolving relationships within Soricinae, some higher-level phylogenetic relationships remain unstable across studies. At the tribal level, the relative placement of Soricini within Soricinae has varied among analytical frameworks [4]. Within Nectogalini, uncertainty also remains regarding the placement of *Pseudosoriculus fumidus* and *Soriculus nigrescens*, as well as the relative position of *Neomys* [4,5,6,7]. These unresolved relationships are especially relevant because Nectogalini includes multiple semi-aquatic lineages, such as *Neomys*, *Chimarrogale*, and *Nectogale*, whose ecological similarity does not always correspond to close phylogenetic relationships. A more complete mitogenomic framework for these lineages is therefore important for distinguishing phylogenetic relatedness from lifestyle-associated similarities and for interpreting mitochondrial genomic variation in an explicit phylogenetic context.

To address this gap, the complete mitogenome of *C. styani* was newly sequenced in this study, a semi-aquatic shrew endemic to Southeast Asia and listed as endangered on China’s Red List of Biodiversity. No complete mitogenome was previously available for this species. Its inclusion increases the representation of semi-aquatic nectogaline shrews and provides a basis for comparing mitochondrial genome evolution between semi-aquatic and terrestrial soricines.

Semi-aquatic soricines differ from terrestrial soricines in several lifestyle-associated traits. They generally have a relatively larger body size within Soricinae [8] and possess morphological features associated with aquatic foraging, including dense fur that can trap air during swimming, partially or fully webbed feet, fringes of stiff hairs along the feet and toes, and elongated terminal phalanges. Although quantitative dietary data are available for only some semi-aquatic soricines, studies of *Neomys* indicate that these species obtain most of their food from terrestrial habitats while deriving approximately 10–20% of their diet from aquatic sources [9]. In several semi-aquatic lineages, additional traits such as ear valves, unpigmented teeth, and modified dental formulae further distinguish them from terrestrial soricines [1,2,3]. These lifestyle-related morphological differences provide an ecological basis for testing whether semi-aquatic and terrestrial soricines also differ in mitochondrial genome evolution.

Mitochondrial genomes are particularly useful for examining lifestyle-associated molecular evolution because they have conserved gene content, relatively rapid evolutionary rates, and central roles in oxidative phosphorylation [10,11]. Ecological transitions that alter energetic demands may therefore leave detectable signatures in mitochondrial genomes, including shifts in nucleotide composition, codon usage, amino-acid properties, and selective constraint. Previous studies of strongly locomotive birds, migratory fish, and cold-adapted fish have shown that mitochondrial genes can experience changes in purifying selection associated with locomotor or environmental demands, presumably reflecting the need to maintain efficient energy production [12,13].

Codon usage provides an additional perspective on mitochondrial genome evolution. It refers to the non-random use of synonymous codons, in which some codons are used more frequently than alternative codons encoding the same amino acid. Such patterns may reflect the combined effects of mutation pressure, natural selection, genetic drift, nucleotide composition, and translational constraints [14,15,16]. In soricines, comparing complete mitogenomes between semi-aquatic and terrestrial lineages can therefore help test whether ecological divergence is associated with differences in mitochondrial composition, synonymous codon usage, amino-acid properties, and selective pressure.

Accordingly, this study aimed to: (1) sequence and characterise the complete mitogenome of *C. styani*; (2) reconstruct a mitochondrial phylogenetic framework for Soricinae with improved sampling of semi-aquatic nectogaline shrews; and (3) test whether semi-aquatic and terrestrial lifestyles are associated with differences in mitochondrial nucleotide composition, codon usage, amino-acid properties, and selective pressure.

## 2. Materials and Methods

### 2.1. Sampling and Identification of C. styani

**Figure 1:**
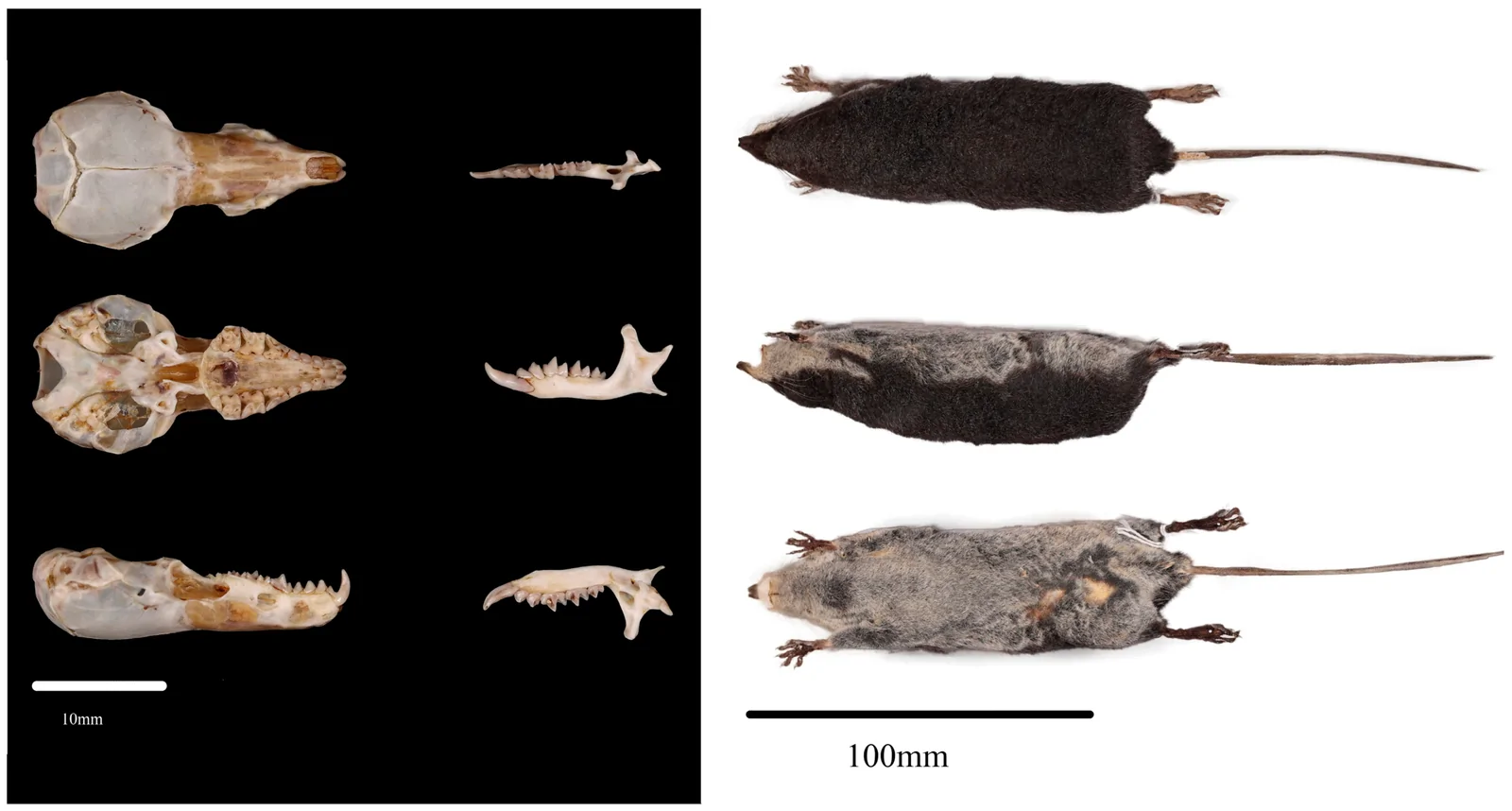
Skull and external morphology of *C. styani*.

**Figure 2:**
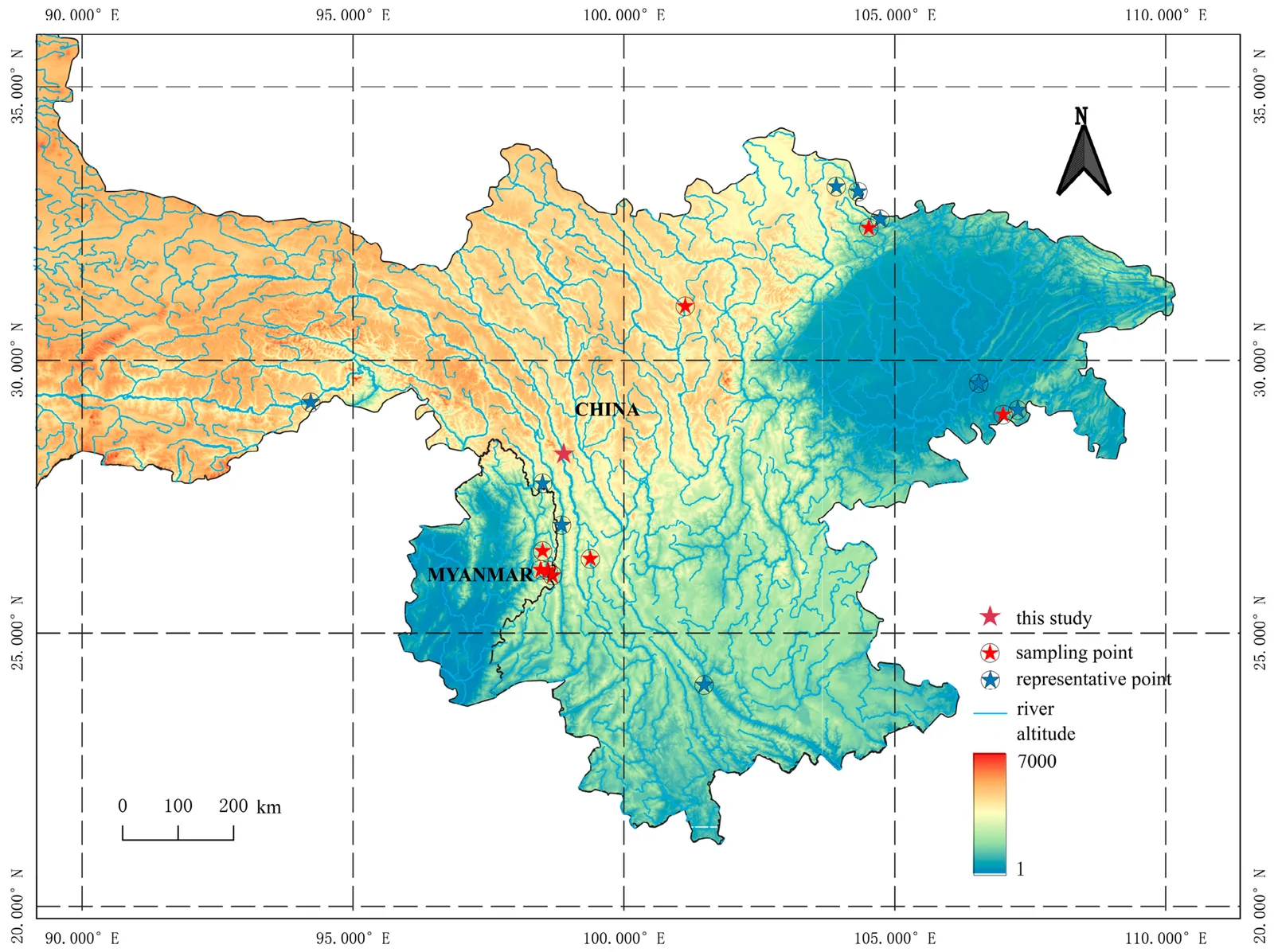
Distribution map of *C. styani.* Solid red stars indicate records from this study; red stars within yellow circles indicate sampling points; blue stars within grey-blue circles indicate representative points from previously known distribution records. Blue lines represent rivers, and the background colour gradient indicates altitude.

A single wild adult male *C*. *styani* (voucher HP2405007) was captured on 10 May 2024 using a shrimp trap in a fast-flowing watercourse at Hongpo Village, Deqin County, Diqing Tibetan Autonomous Prefecture, Yunnan Province, China (28°16′50.08″ N, 98°53′20.01″ E; 2400 m a.s.l.). Standard morphometric measurements were recorded from the fresh specimen in the field, including head–body length (100 mm), tail length (90 mm), hindfoot length (21 mm), ear length (5 mm), and body mass (22.5 g). The voucher specimen was deposited in the Institute of Pathogens and Vectors, Dali University. Muscle, liver, and spleen tissue samples were collected from the same individual under sterile conditions, preserved in 95% ethanol, and stored at −80 °C until subsequent analysis. Morphological identification was conducted based on diagnostic characters described in *Handbook of the Mammals of the World* [2] and *The Mammals of Sichuan* [3]. The specimen showed diagnostic features consistent with *C. styani*. It had a long, pointed snout and a uniformly dark grayish-black dorsum extending from the snout tip to the tail base. Scattered white guard hairs were present on the dorsum but absent from the head, and were especially long and conspicuous on the rump. The venter was white, with hairs slightly pale yellow at the tips and grey at the bases, forming a distinct boundary between the dorsal and ventral colouration. Other diagnostic features included a bicoloured tail, partially webbed feet, and fringes of stiff hairs along the margins of the feet and toes. Its skull was small, flat, broad, and elongated, with thin cranial bones, and the teeth were unpigmented (Figure 1). For species identification, BLAST (https://blast.ncbi.nlm.nih.gov/Blast.cgi, accessed on 30 September 2026) searches were performed using the complete *CYTB* sequence obtained from this sample. The mitochondrial *CYTB* fragment was first amplified using the primers H15915_hk3 and L14724_hk3 [17]. PCR amplification was performed with an annealing temperature of 49 °C for 35 cycles. The assembled fragment was 1171 bp in length, including the complete 1140 bp *CYTB* coding region. Standard Nucleotide BLAST identified *C. styani* KC794773.1, with 99.56% identity and 97% query coverage. Other homologous sequences with high similarity were KC794772.1 (99.30% identity) and MW682661.1 (99.47% identity). Sequences from closely related *Chimarrogale* congeners were also retrieved and examined during the BLAST comparison. No congeneric sequence showed a higher similarity than the identified *C. styani* matches, supporting the assignment of the specimen as *C. styani*. All experimental procedures involving *C. styani* were reviewed and approved by the Laboratory Animal Ethics Committee of Dali University (approval code: 2021-P2-162; approval date: 10 January 2021). *C. styani* is an endemic species to Southeast Asia and has been listed as an endangered species on China’s Red List of Biodiversity. A species distribution map was generated using QGIS 3.40.14 based on occurrence records compiled from the published literature, museum collections, and online databases (QGIS Development Team, https://qgis.org/; accessed on 16 September 2026) (Figure 2 and Table S1).

### 2.2. Sequencing, Assembly and Annotation

Shenzhen Huitong Biotechnology Co., Ltd. (Shenzhen, China) performed library preparation, sequencing, and preliminary bioinformatic processing for the voucher specimen HP2405007. The integrity of extracted genomic DNA was evaluated by 1% agarose gel electrophoresis, and DNA concentration was quantified using a Qubit^®^ 3.0 Fluorometer and the Qubit^®^ DNA Assay Kit (Thermo Fisher Scientific, Waltham, MA, USA). A single genomic DNA library (library ID: FDSW250351772-1r) was constructed from specimen HP2405007. The DNA sample was randomly fragmented to approximately 350 bp using a Covaris ultrasonicator (Covaris, Inc., Woburn, MA, USA), followed by end repair, A-tailing, adapter ligation, fragment selection, PCR amplification, and purification using the NEBNext^®^ Ultra™ DNA Library Prep Kit for Illumina (New England Biolabs, Ipswich, MA, USA). The library was sequenced using an Illumina platform (Illumina, Inc., San Diego, CA, USA) to generate 150 bp paired-end reads. The HP2405007 library generated 33,137,700 raw reads (4.97 Gb). After quality filtering, 32,803,786 clean reads (4.92 Gb) were retained, with an effective rate of 98.99%. The sequencing data showed Q20 and Q30 values of 98.73% and 95.31%, respectively. The mitochondrial genome assembly was performed by Shenzhen Huitong Biotechnology Co., Ltd. using their standard mitochondrial genome assembly pipeline. The resulting assembly was subsequently visualised and manually inspected using Bandage [18], and read mapping was performed using BWA to validate the assembly.

**Figure 3:**
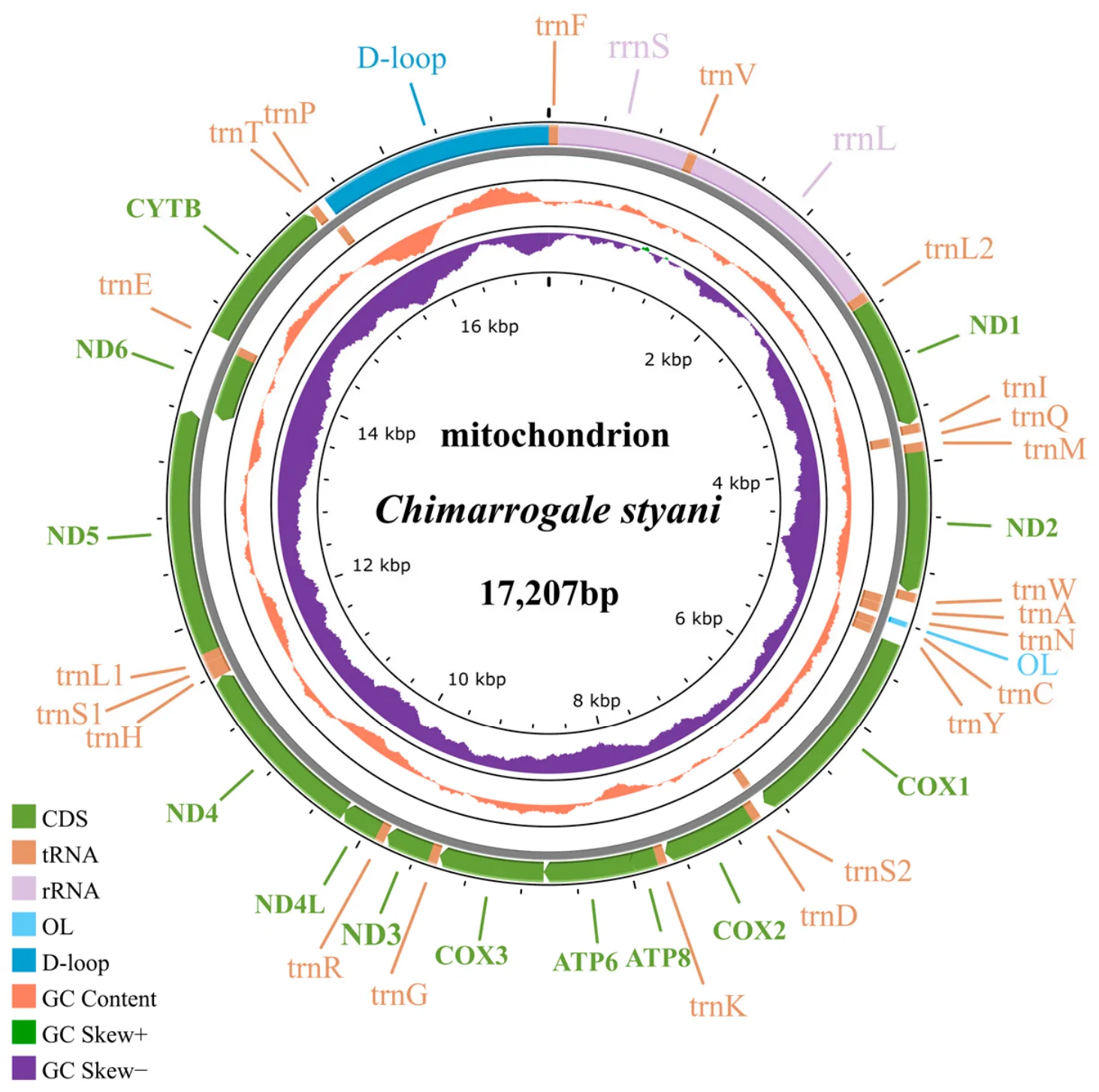
The circular map of the *C. styani* mitogenome. Genes are colour-coded by functional category: protein-coding genes (green), transfer RNAs (brown), ribosomal RNAs (purple), and the control region (blue).

To verify the preliminary assembly, the complete mitogenome of *C. styani* was independently reassembled and checked in Geneious Prime v11.1.5 [19]. Thirteen protein-coding genes (PCGs), 22 transfer RNAs, and 2 ribosomal RNAs were initially annotated using MITOS2 (https://usegalaxy.eu/root?tool_id=toolshed.g2.bx.psu.edu/repos/iuc/mitos2/mitos2/; accessed on 16 September 2026) [20] with the vertebrate mitochondrial genetic code. All predicted gene boundaries were further verified by sequence alignment with closely related soricine mitogenomes from NCBI. The secondary structures of 22 tRNA genes were confirmed with tRNAscan-SE v2.0 [21], and all annotation inconsistencies were manually corrected. The newly sequenced mitogenome sequence of *C. styani* has been deposited in GenBank under accession number PX068317. The circular genome map (Figure 3) was visualised with Proksee (https://proksee.ca/; accessed on 16 September 2026) [22].

### 2.3. Phylogenetic Reconstruction

The complete mitogenome of *C. styani* was newly sequenced in this study. The newly sequenced *C. styani* mitogenome was combined with 72 complete mitogenomes of Soricidae obtained from NCBI, with *Uropsilus gracilis* and *Uropsilus investigator* designated as outgroups. Phylogenetic analyses were conducted to clarify the mitochondrial placement of *C. styani* within Soricinae, assess relationships among major soricine lineages, and provide a framework for comparing mitochondrial evolution between semi-aquatic and terrestrial soricines. Protein-coding genes and rRNA sequences were aligned at the nucleotide level using MAFFT v7.5.5 [23]. PCG alignments were further refined with MACSE v2.06 [24], which uses amino-acid translation to guide nucleotide alignment while maintaining codon structure, and trimmed with Gblocks v0.91b [25] in codon mode. The following Gblocks parameters were applied: minimum number of sequences for a conserved position = 35, minimum number of sequences for a flank position = 35, maximum number of contiguous nonconserved positions = 8, minimum block length = 10, and allowed gap positions = with half. rRNA sequences were trimmed using trimAl v1.2rev57 [26] with the automated1 option. The final concatenated alignment consisted of 13 protein-coding genes and two rRNA genes. Substitution saturation of the PCGRNA alignment was evaluated using the Xia test in DAMBE v7.3.32 [27]. The index of substitution saturation (Iss) was lower than the critical value (Iss.c) (Figure S1), supporting the suitability of the dataset for subsequent phylogenetic reconstruction. The optimal partitioning strategy and nucleotide substitution model were determined using ModelFinder v2.2.0 [28]. Phylogenetic relationships were inferred using two approaches: (1) Bayesian inference (BI) in MrBayes v3.2.7 [29], with four independent MCMC runs of 5,000,000 generations, sampling every 1000 generations and discarding the initial 25% as burn-in; convergence was confirmed by ASDSF < 0.01, PSRF values close to 1.00, and ESS values > 200; and (2) maximum-likelihood (ML) analysis in IQ-TREE v2.2.0 [30], with branch support evaluated using 5000 ultrafast bootstrap replicates. The consensus trees were visualised and edited using tvBOT (https://www.chiplot.online/tvbot.html, accessed on 16 September 2026) [31].

### 2.4. Comparative Mitogenomic Analyses of Semi-Aquatic and Terrestrial Soricines

Lifestyle categories were assigned according to published ecological and taxonomic descriptions [2,3,4]. Species consistently described as aquatic or semi-aquatic were classified as semi-aquatic, whereas primarily ground-dwelling species without specialized aquatic habits were classified as terrestrial. Species with ambiguous lifestyle information were excluded. Consequently, 11 species with both complete mitogenomes and clearly documented lifestyles were retained for comparative analyses (Table 1). The AT content (AT%), AT-skew, and GC-skew were calculated using PhyloSuite v1.2.3 [32]. Skewness values were calculated using the standard formulas: AT-skew = [A − T]/[A + T] and GC-skew = [G − C]/[G + C]. Codon usage bias was evaluated for the 13 PCGs using CodonW v1.4.2 (https://sourceforge.net/projects/codonw/; accessed on 16 September 2026). Codon usage bias refers to the non-random use of synonymous codons, and several indices were used to quantify different aspects of this bias. The nucleotide frequencies at the third codon position (A3s, T3s, C3s, G3s, and GC3s) and the GC content at the first and second codon positions (GC12) were computed using a Python script (Python v3.12.7). The codon adaptation index (CAI) [33] measures the extent to which the codon usage of a gene resembles that of a reference set of highly expressed genes. CAI ranges from 0 to 1, with higher values indicating greater conformity to the codon-usage pattern of highly expressed genes and potentially stronger translational optimisation. The codon bias index (CBI) [34] measures the degree to which codon usage is biased toward preferred codons. A CBI value of 1 indicates exclusive use of preferred codons, whereas a value of 0 indicates random codon choice. Negative values may occur when non-preferred codons are used more frequently than expected by chance. The F_op_ [35] represents the frequency of optimal codons among synonymous codons, with values ranging from 0.0 (no bias) to 1.0 (maximum bias). As a key indicator of codon usage bias, relative synonymous codon usage (RSCU) reflects the relative probability of a given codon encoding its corresponding amino acid among synonymous codons [36]. To characterise amino-acid composition and protein properties, the grand average of hydropathicity (GRAVY) and aromaticity (Aromo) were also calculated. Gravy reflects the average hydrophobicity of the encoded proteins, with positive and negative values indicating hydrophobic and hydrophilic tendencies, respectively. Aromo represents the relative frequency of aromatic amino acids, including phenylalanine, tyrosine, and tryptophan. Differences in nucleotide composition and codon-usage indices between semi-aquatic and terrestrial soricines were tested using Mann–Whitney U tests with false discovery rate correction.

The ENC-GC3s plot was constructed to assess the factors driving codon usage bias across different lifestyles, with the effective number of codons (ENC) as the ordinate and GC3s as the abscissa. The standard curve, calculated using the equation ENC_{expected} = 2 + s + 29/[s^2^ + (1 − s)^2^], where s represents GC3s [37], represented theoretical ENC values driven solely by mutational pressure. Genes distributed along or near the standard curve were interpreted as indicating that codon usage bias was dominated by mutation, whereas genes that fell below and further away from the standard curve were interpreted as suggesting the dominant influence of natural selection. Parity rule 2 (PR2) analysis was used to calculate the ratios A3s/(A3s + T3s) and G3s/(G3s + C3s). In the PR2 plot, the central point (A = T, C = G) represented no selection pressure on codon usage [38]. Correspondence analysis (COA) was applied to examine codon usage bias across different lifestyles and explore divergent evolutionary history and selective pressures [39]. Neutrality-curve analysis was used to assess the relative contributions of natural selection and mutational pressure to codon usage bias, with GC12 as the ordinate and GC3s as the abscissa. The diagonal distribution (regression coefficient ≈ 1) indicated mutational dominance, while a scattered distribution (regression coefficient ≈ 0) signified the predominance of natural selection [40]. ENC-GC3s plot, neutrality curve, PR2 plot, and COA were visualised using RStudio [41], and permutational multivariate analysis of variance (PERMANOVA) was used to assess differences between the two groups. For each species, the nonsynonymous substitution rate (Ka) and synonymous substitution rate (Ks) for the 13 PCGs were calculated using KaKs Calculator 3.0 [42], and Between-group comparisons of Ka and Ks were conducted using Mann–Whitney U tests with FDR correction.

### 2.5. Phylogenetic Comparative Methods

To account for phylogenetic non-independence among species, phylogenetic generalized least-squares (PGLS) analyses were performed using the mitochondrial phylogeny inferred in this study. The phylogeny was pruned to the 11 species included in the comparative analyses while retaining branch lengths. Lifestyle was treated as a categorical predictor, with terrestrial soricines used as the reference group. Pagel’s λ was estimated by maximum likelihood using a profile grid ranging from 0 to 1 at intervals of 0.01. Separate PGLS models were fitted for mitochondrial genome size, nucleotide-composition variables, codon-usage indices, amino-acid properties, and PR2 coordinates. For the 13 mitochondrial protein-coding genes, Ka/Ks ratios were analysed separately using gene-specific PGLS models. *p* values were adjusted using the Benjamini–Hochberg false-discovery-rate procedure within each family of comparisons. To further evaluate the neutrality and ENC–GC3s relationships while accounting for phylogeny, interaction models including GC3s, lifestyle, and their interaction were fitted for GC12 and ENC, respectively. PGLS analyses were conducted in R using the ape and nlme packages.

## 3. Results

### 3.1. Morphology and Mitogenome of C. styani

The complete mitogenome of *C. styani* is 17,207 bp long and exhibits a highly conserved gene arrangement pattern typical of vertebrate mitochondrial genomes [10,11] (Figure 3). It contains 37 mitochondrial genes (13 PCGs, 2 rRNAs, and 22 tRNAs) and one control region. Nine genes (*trnQ*, *trnA*, *trnN*, *trnC*, *trnY*, *trnS_2_*, *ND6*, *trnE*, and *trnP*) are encoded on the light strand, whereas the remaining 28 genes are encoded on the heavy strand. No gene losses, duplications, or rearrangements were detected across the entire mitogenome, suggesting an overall stable and broadly conserved genomic organisation consistent with most vertebrate mitogenomes. A total of six overlapping regions of variable sizes (1–43 bp) and 14 intergenic spacer regions (1–6 bp) were identified in the *C. styani* mitogenome. The largest overlap (43 bp) occurs between *ATP8* and *ATP6*, a common overlapping feature in vertebrate mitogenomes. The largest intergenic spacer region (6 bp) was found between *trnS_2_* and *trnD*. The nucleotide composition of the *C. styani* mitogenome consists of A (33.3%), T (31.4%), G (12.5%), and C (22.8%), with a total AT content of 64.7% and a GC content of 35.3%. The AT-skew value was 0.03, indicating that adenine was only slightly more frequent than thymine within the same mitogenome. The GC-skew value was −0.293, indicating a clear excess of cytosine over guanine. The total length of 13 PCGs is 11,415 bp, accounting for 66.3% of the entire mitogenome. For these PCGs, the overall AT and GC contents are 64.6% and 35.4%, with corresponding AT-skew and GC-skew values of −0.022 and −0.305, respectively. The AT content of the 13 protein-coding genes ranges from 60.9% to 74%, with *CYTB* exhibiting the lowest (60.9%) and ATP8 showing the highest (74%). All start codons for 13 protein-coding genes are ATN. However, the stop codons are variable among different genes: *COX1*, *COX2*, *ATP8*, *ATP6*, *ND3*, *ND4L*, *ND5*, and *ND6* use TAN stop codons; *CYTB* uses the uncommon AGA stop codon; and four remaining genes (*ND1*, *ND2*, *COX3*, and *ND4*) possess an incomplete stop codon (T).

### 3.2. Phylogenetic Relationships

**Figure 4:**
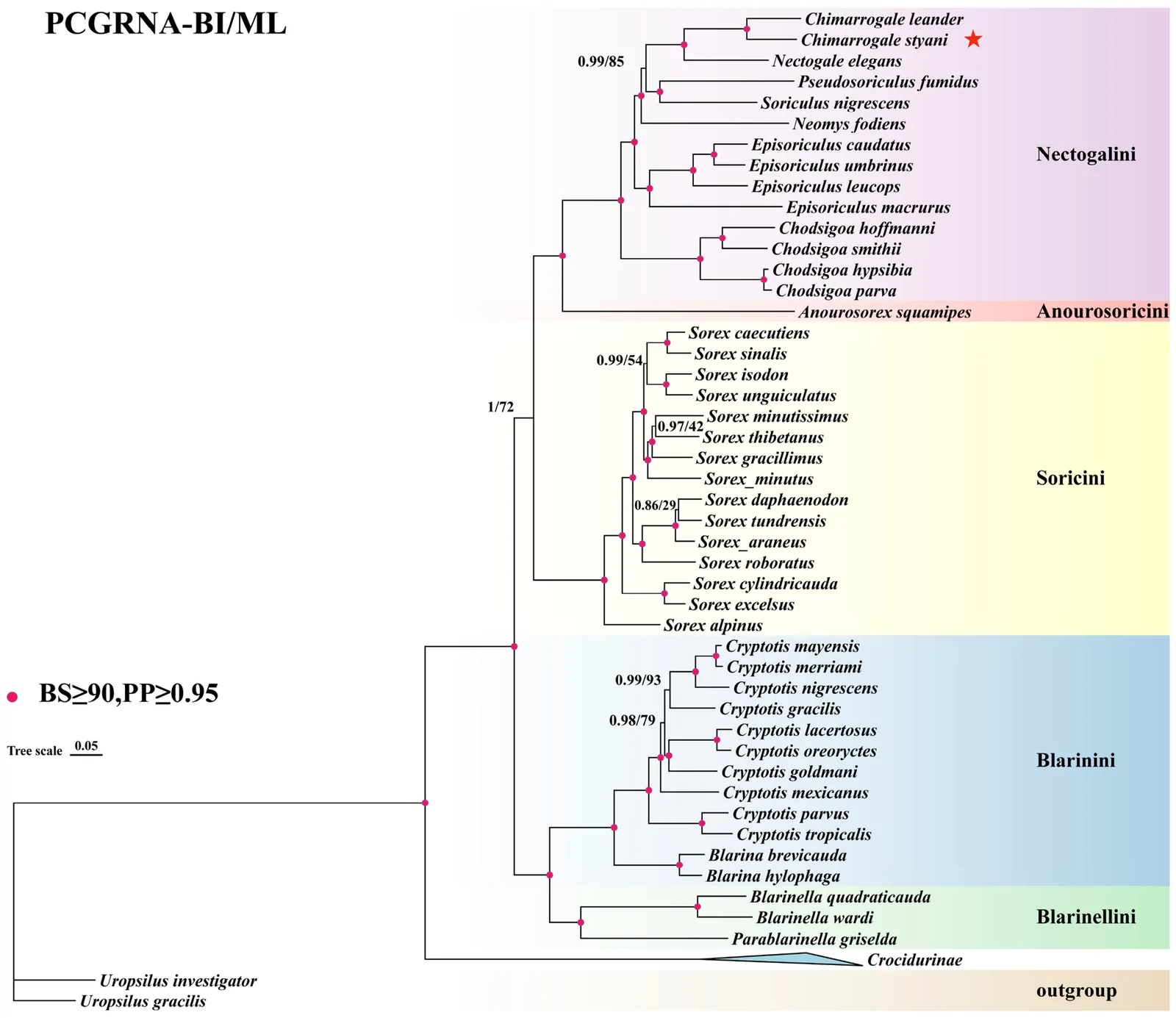
Phylogenetic relationships within Soricinae inferred from the PCGRNA alignment comprising 13 protein-coding genes and two ribosomal RNA genes using Bayesian inference and maximum-likelihood methods, with *U. gracilis* and *U. investigator* as outgroups. Solid red dots at nodes indicate strong support in both analyses (ML bootstrap support, BS ≥ 90%; Bayesian posterior probability, PP ≥ 0.95). The red star indicates the species sequenced in this study.

Complete mitogenome sequences of Soricidae were downloaded from NCBI. These sequences were combined with the *C. styani* mitogenome newly sequenced in this study. *U. gracilis* and *U. investigator* were used as outgroups. A PCGRNA alignment was constructed from 13 PCGs and 2 rRNA genes. This indicates low substitution saturation and confirms the suitability of the dataset for phylogenetic reconstruction. Phylogenetic trees reconstructed using ML and BI recovered identical topologies (Figure 4) and strongly supported the monophyly of Soricinae (ML bootstrap support (BS) = 100%; BI posterior probability (PP) = 1.00). However, phylogenetic relationships within several tribes remained partially unresolved, as indicated by weaker branch support values at certain internal nodes. The lack of resolution may be due to limited or insufficient taxon sampling within these tribes. Within the semi-aquatic soricines, Chimarrogale was recovered as sister to Nectogale with strong support (BS = 100%; PP = 1). *C. leander* and *C. styani* formed one strongly supported clade, which itself clustered with *N. elegans*.

### 3.3. Comparative Mitogenomic Patterns in Semi-Aquatic and Terrestrial Soricines

The mitogenomes of the semi-aquatic soricines in this study ranged from 16,173 to 17,460 bp in length. The average lengths of the 13 PCGs varied from ATP8 (204 bp) to ND5 (1821.75 bp), whereas the 22 tRNA genes had average lengths ranging from trnS1 (59 bp) to trnL2 (75 bp). The rrnS and rrnL genes spanned 963–968 bp and 1564–1578 bp, respectively, and the control region ranged from 681 bp to 1989 bp. In contrast, the mitogenomes of terrestrial soricines ranged from 15,298 to 17,444 bp in length. Their 13 PCGs exhibited a similar range of average lengths, from 204 bp (ATP8) to 1821 bp (ND5), and their 22 tRNA genes averaged between trnS1 (59 bp) and trnL2 (75 bp). The lengths of rrnS (665–968 bp) and rrnL (1563–1591 bp) genes were similarly conserved, whereas the control region was more variable (198 bp–1783 bp). Overall, mitogenome sizes differed only slightly between semi-aquatic and terrestrial soricines, and the lengths of all 37 encoded genes exhibited no substantial differences.

**Figure 5:**
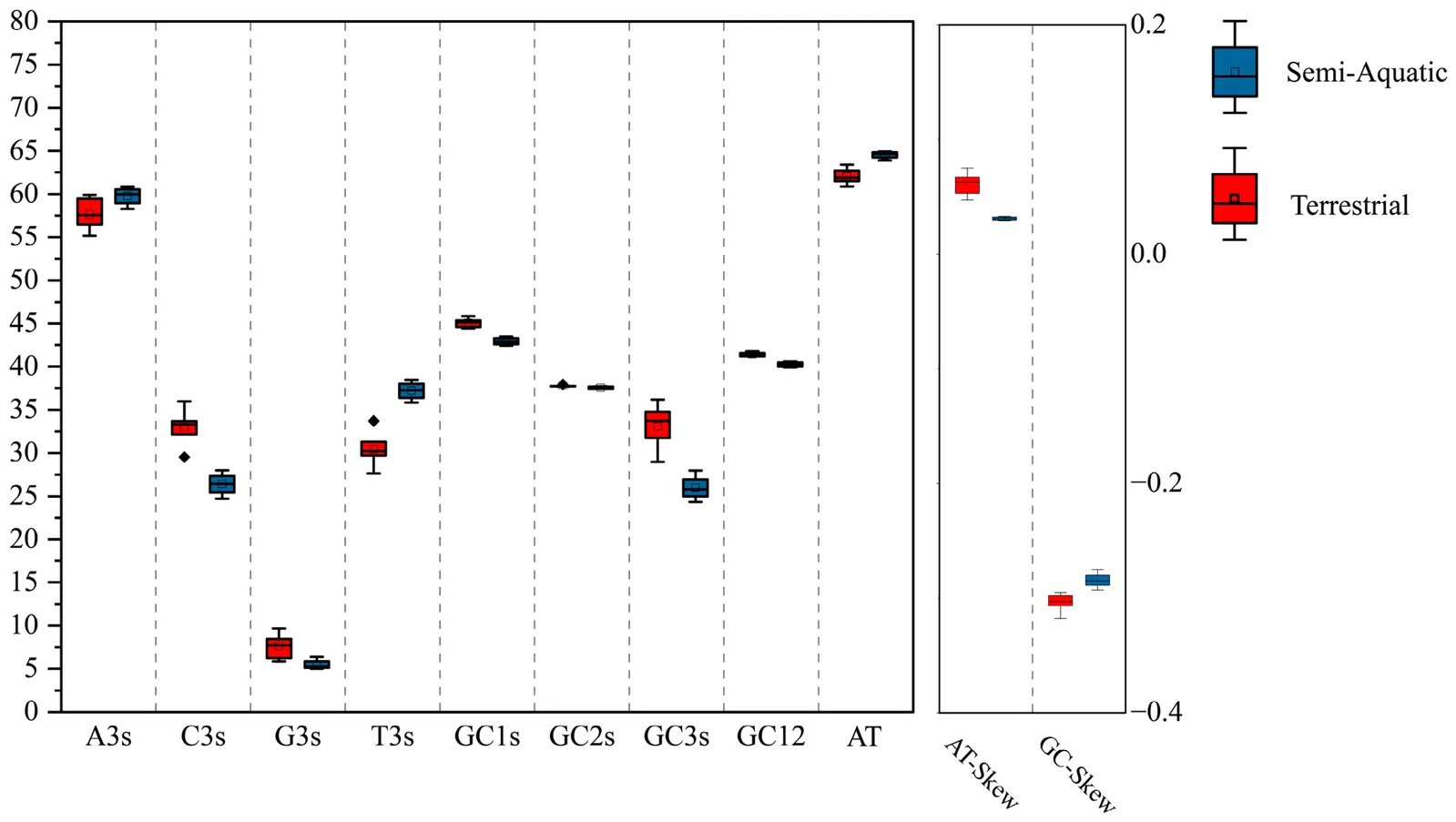
Comparison of nucleotide composition between semi-aquatic and terrestrial soricines. Bars represent mean values, and error bars indicate standard deviations. The variables shown include A3s, C3s, G3s, T3s, GC1s, GC2s, GC3s, GC12, AT content, AT-skew, and GC-skew. Formal phylogenetically controlled comparisons were performed using PGLS and are reported in Section 3.4 and Table S4. Black diamonds indicate outliers.

The mean T3s, C3s, A3s, G3s, GC1s, GC2s, GC3s, and GC12 values were calculated for terrestrial soricines (Figure 5), with values of 30.43%, 33.00%, 57.64%, 7.63%, 45.04%, 37.77%, 33.09%, and 41.44%, respectively; for semi-aquatic soricines, the corresponding values were 37.21%, 26.40%, 59.76%, 5.49%, 42.93%, 37.55%, 25.96%, and 40.25%, respectively. The mean AT content was higher in semi-aquatic soricines (64.525%) than in terrestrial soricines (62.1%). The mean AT-skew was also lower in semi-aquatic soricines (0.031) than in terrestrial soricines (0.061). In contrast, the mean GC-skew was negative across eleven species examined and was more negative in terrestrial soricines (−0.304) than in semi-aquatic soricines (−0.285). Mann–Whitney U tests with FDR correction further revealed significant differences in nucleotide composition between the two groups.

Terrestrial soricines showed slightly higher codon adaptation index values than semi-aquatic soricines (0.14 vs. 0.13), indicating that their mitochondrial protein-coding genes more closely matched the reference codon-usage pattern. Terrestrial soricines also showed a less negative codon bias index (−0.066 vs. −0.122) and a higher frequency of optimal codons (0.376 vs. 0.343). The latter value means that approximately 37.6% of the relevant synonymous codons in terrestrial soricines, compared with 34.3% in semi-aquatic soricines, were classified as preferred or optimal according to the reference codon set. Together, these results suggest a modestly stronger bias toward preferred synonymous codons in terrestrial soricines.

**Figure 6:**
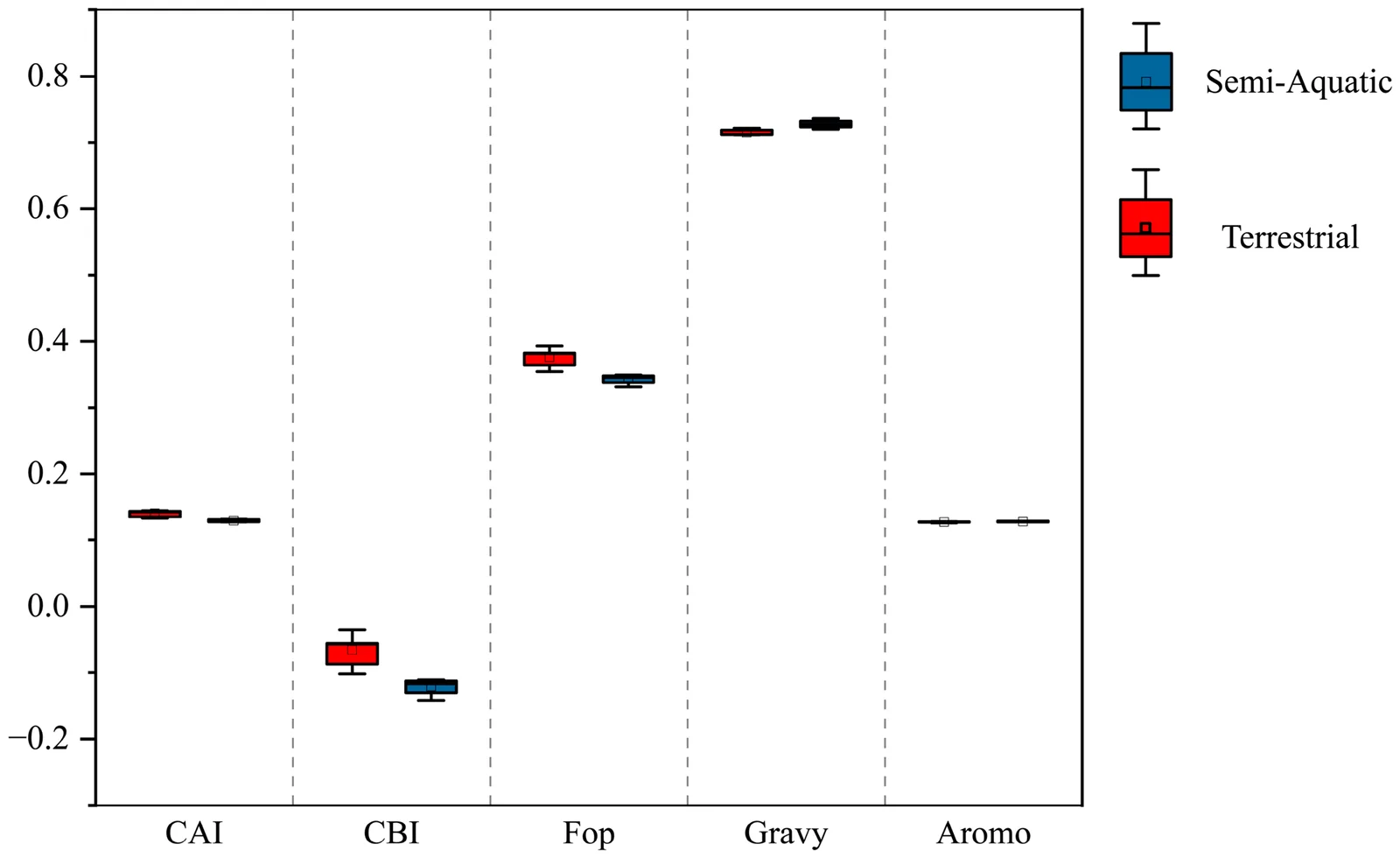
Comparison of codon-usage and protein-property indices between semi-aquatic and terrestrial soricines. Box plots show the distributions of the codon adaptation index (CAI), codon bias index (CBI), frequency of optimal codons (Fop), grand average of hydropathicity (GRAVY), and aromaticity (Aromo). Black squares indicate mean values, and horizontal lines within the boxes indicate medians. Formal phylogenetically controlled comparisons were performed using PGLS and are reported in Section 3.4 and Table S4.

In contrast, the grand average of hydropathicity values was positive in all species, indicating that the encoded mitochondrial proteins were generally hydrophobic. Mean hydropathicity was slightly higher in semi-aquatic soricines than in terrestrial soricines (0.728 vs. 0.715), suggesting slightly greater average hydrophobicity of mitochondrial proteins in the semi-aquatic group. Among the 13 mitochondrial protein-coding genes, only *ATP8* showed a negative hydropathicity value, indicating a relatively hydrophilic protein. Aromaticity, which measures the relative frequency of aromatic amino acids, did not differ significantly between semi-aquatic and terrestrial soricines (0.1278 vs. 0.1270). Mann–Whitney U tests followed by false discovery rate correction showed significant differences for all measured indices except aromaticity (Figure 6).

**Figure 7:**
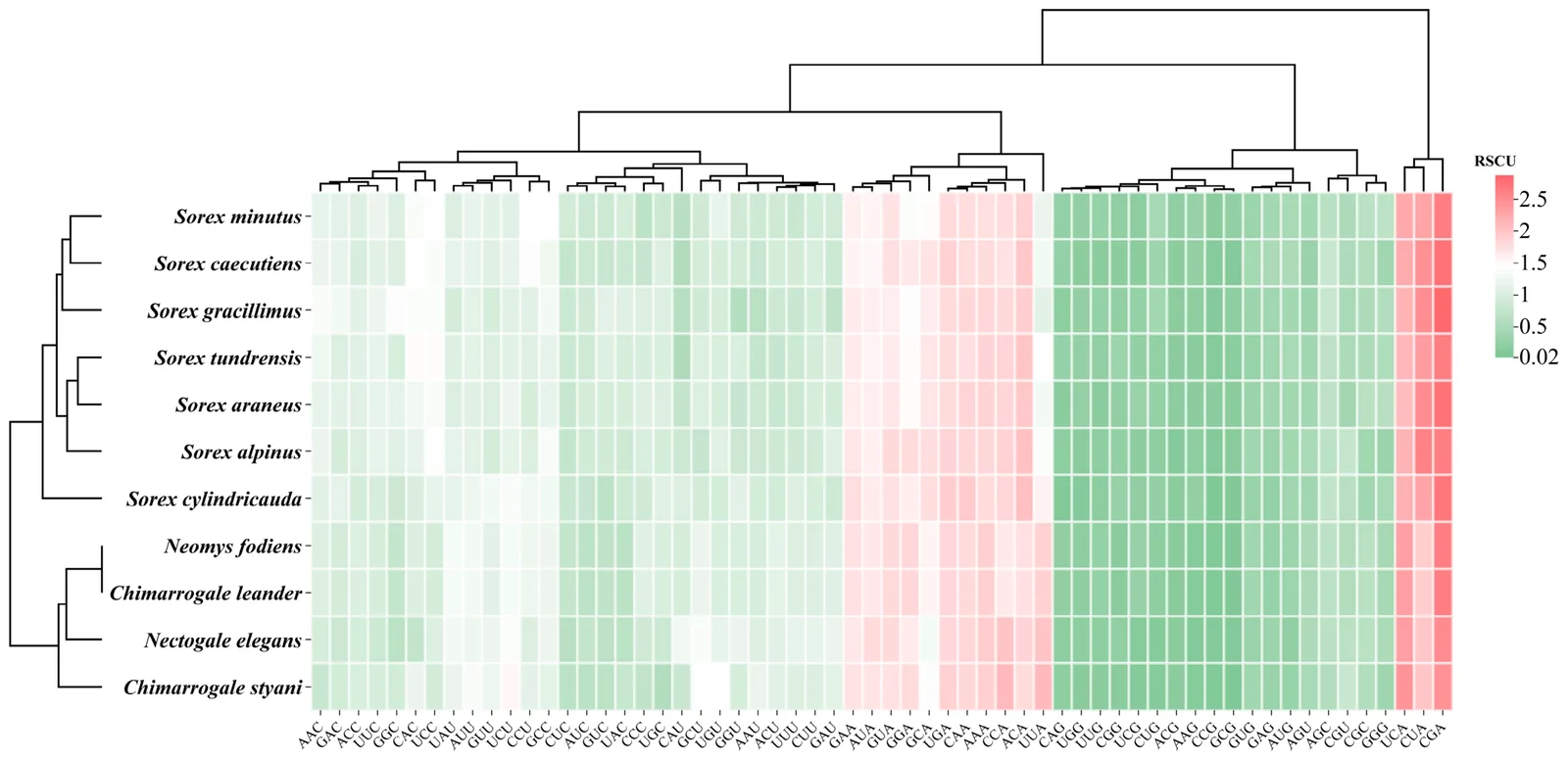
Clustering heatmap of semi-aquatic and terrestrial shrew species based on relative synonymous codon usage (RSCU). Colour intensity reflects RSCU values; red indicates high usage, green indicates low usage.

The RSCU-based heatmap revealed distinct patterns of synonymous codon usage among the sampled soricines (Figure 7). Hierarchical clustering grouped species with similar RSCU profiles and grouped codons with similar variation across species. The number of preferred codons (RSCU > 1) ranged from 27 to 31 in terrestrial soricines and from 29 to 31 in semi-aquatic soricines. Notably, all semi-aquatic soricines shared the same 13 overrepresented codons (RSCU > 1.6), whereas terrestrial soricines exhibited greater variation, with the number ranging from 12 to 14. All overrepresented codons ended in adenine (A). CGA was the most frequently used codon across all examined species.

**Figure 8:**
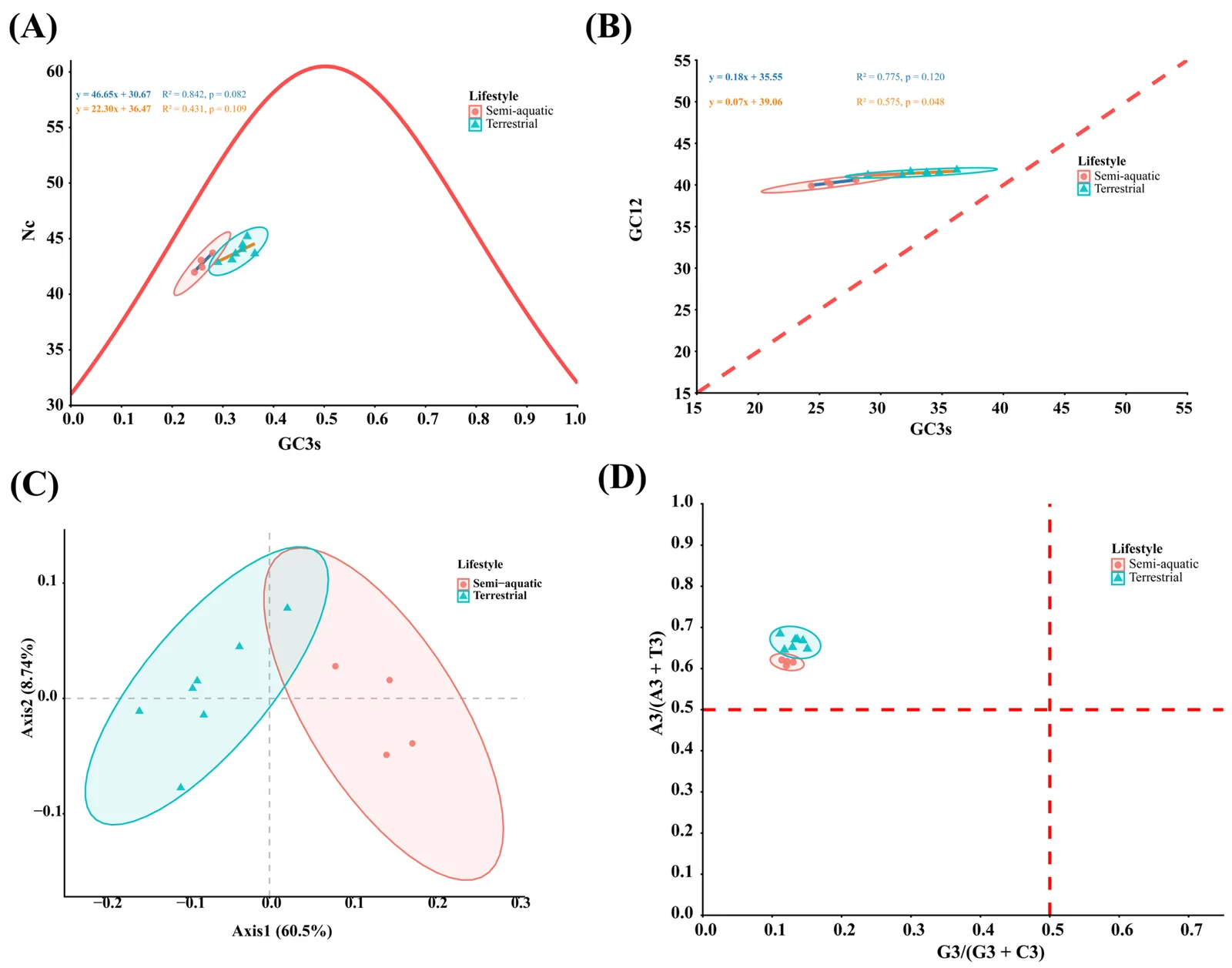
Multivariate analyses of codon usage bias for 13 mitochondrial protein-coding genes in semi-aquatic and terrestrial soricines: (**A**) effective number of codons (ENC) plot; (**B**) neutrality curve; (**C**) correspondence analysis (COA); and (**D**) parity rule 2 (PR2) plot. Colours distinguish semi-aquatic and terrestrial soricines as indicated in the legends. In (**A**), the red solid curve represents the theoretical expected ENC curve under mutation-only codon usage. In (**B**), the red dashed diagonal represents the neutrality reference line GC12 = GC3s, whereas the coloured lines show the fitted relationships for the two lifestyle groups. In (**C**), the coloured ellipses illustrate the distributions of the two lifestyle groups in ordination space. In (**D**), the red dashed lines indicate the parity reference values of 0.5 for both axes, and the coloured ellipses show the distributions of the two lifestyle groups.

ENC and PR2 analyses were performed for the 13 mitochondrial protein-coding genes of each species. The effective number of codons (ENC) quantifies codon usage bias, with lower values indicating stronger bias. The ATP8 gene lay above the standard curve for all sampled soricines (Figures S2 and S3), with ENC values ranging from 45.6 to 61, indicating generally weak codon usage bias. In the PR2 analysis, ATP8 was positioned closer to the central point (A = T, G = C) than most other genes (Figures S4 and S5). ENC, PR2, COA, and neutrality-curve analyses were further performed on the concatenated protein-coding genes of the eleven species. In the ENC plot (Figure 8A), all points fell below the expected curve. Neutrality-curve analysis (Figure 8B) showed descriptive regression slopes of 0.18 and 0.07 for semi-aquatic and terrestrial soricines, respectively. COA (Figure 8C) showed separation between the sampled ecological groups in ordination space, while PR2 analysis (Figure 8D) showed all eleven species in the upper-left quadrant, indicating A/T and C/G imbalance at synonymous third codon positions. Formal phylogenetically controlled comparisons of these patterns are reported below.

**Figure 9:**
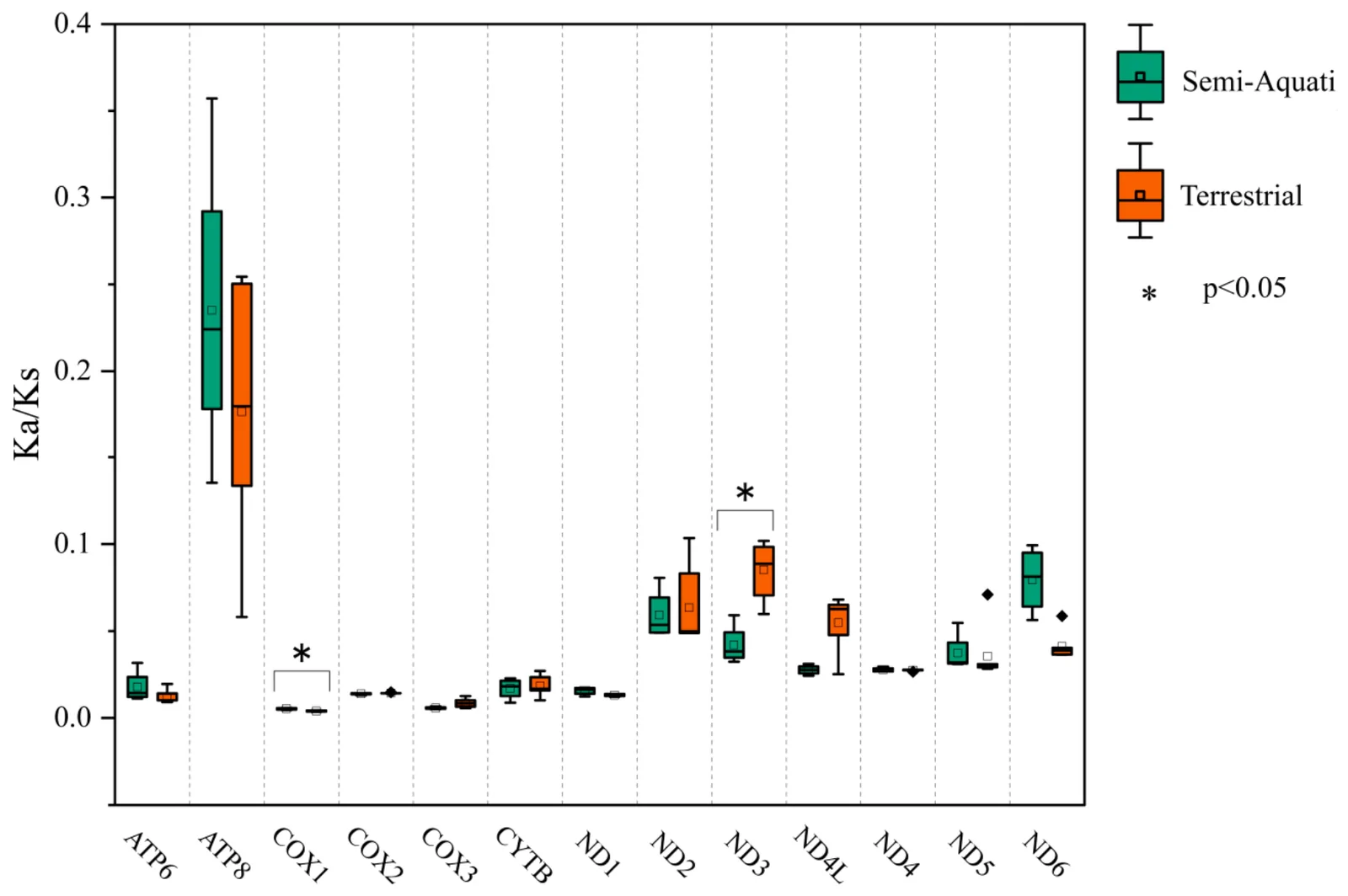
Selective pressure analysis of protein-coding genes between semi-aquatic and terrestrial soricines. Boxes indicate the interquartile range, horizontal lines within boxes indicate medians, square markers indicate mean values, whiskers show the data range excluding outliers, and black diamonds indicate outliers. Bars represent Ka/Ks ratios; asterisks indicate significant differences (*p* < 0.05, Mann–Whitney U test with FDR correction). Black diamonds indicate outliers.

The Ka/Ks ratios of all 13 PCGs were below 1 in both semi-aquatic and terrestrial soricines (Figure 9), indicating pervasive purifying selection. Descriptively, *ATP8*, *ATP6*, *COX1*, *ND1*, *ND4*, *ND5*, *ND6*, and *CYTB* showed higher mean Ka/Ks ratios in semi-aquatic soricines, whereas *COX2*, *COX3*, *ND2*, *ND3*, and *ND4L* showed higher mean ratios in terrestrial soricines. Formal gene-specific PGLS comparisons are reported below.

### 3.4. Phylogenetically Controlled Comparative Analyses

PGLS analyses showed that most of the major nucleotide-composition differences between semi-aquatic and terrestrial soricines remained significant after accounting for phylogenetic relationships and multiple-testing correction. Semi-aquatic soricines had significantly higher AT content and T3s values and significantly lower C3s, G3s, GC1s, GC2s, GC3s, and GC12 values (all q < 0.05). AT-skew was significantly lower, whereas GC-skew was significantly less negative in semi-aquatic soricines. A3s was higher in semi-aquatic soricines, but this difference did not remain significant after FDR correction (q = 0.061).

Codon-usage analyses also retained several significant lifestyle-associated differences after phylogenetic correction. Terrestrial soricines had significantly higher CAI, CBI, and Fop values, whereas Gravy was significantly higher in semi-aquatic soricines. In contrast, aromaticity and concatenated ENC did not differ significantly between lifestyles after FDR correction.

The numbers of preferred codons (RSCU > 1) and strongly overrepresented codons (RSCU > 1.6) did not differ significantly between lifestyles after FDR correction. In the phylogenetically corrected neutrality model, GC12 was significantly associated with GC3s (*p* = 0.039), but neither the lifestyle effect nor the GC3s × lifestyle interaction was significant (*p* = 0.116 and *p* = 0.171, respectively). Similarly, the ENC–GC3s model detected no significant lifestyle effect or GC3s × lifestyle interaction.

Gene-specific PGLS analyses of Ka/Ks identified significant lifestyle-associated differences in four mitochondrial genes after FDR correction. *COX1* and *ND6* had significantly higher Ka/Ks ratios in semi-aquatic soricines, whereas *ND3* and *ND4L* had significantly lower ratios. No significant differences were detected for the remaining nine genes after multiple-testing correction.

## 4. Discussion

### 4.1. Phylogenetic Placement and Relationships

The phylogenetic analyses, based on the PCGRNA alignment, strongly supported the monophyly of Soricinae (BS = 100%; PP = 1.00) and recovered five major tribes: Nectogalini, Blarinini, Soricini, Blarinellini, and Anourosoricini. Notiosoricini was not included because complete mitogenomic data were unavailable. Maximum-likelihood and Bayesian analyses yielded identical topologies, supporting the reliability of the mitochondrial signal for the sampled taxa. Within Nectogalini, the placement of *C. styani* with *C. leander* supports the mitochondrial affinity of the newly sequenced species and confirms the close relationship between *Chimarrogale* and *Nectogale*. Although *Neomys fodiens* also has a semi-aquatic lifestyle, it was not recovered as sister to the *Chimarrogale* + *Nectogale* clade in the present tree, which is consistent with previous studies [4,5,6,7,43,44,45,46,47]. This pattern is consistent with He et al. [4], who suggest that semi-aquatic adaptations in Nectogalini may have evolved repeatedly or convergently rather than from a single semi-aquatic ancestor.

The placement of several nectogaline taxa nevertheless remains unstable among studies [5,43,44,45,46]. In the mitogenomic tree, *P. fumidus* clustered with *S. nigrescens*, a result consistent with its exclusion from *Episoriculus*. This agrees with recent integrative work by Liu et al. [47], who combined mitochondrial, nuclear, and morphological evidence and transferred *P. fumidus* to *Pseudosoriculus*. However, Liu et al. [47] also showed that the precise placement of *P. fumidus* within Nectogalini varies among datasets: it clustered with *Chodsigoa* in the *CYTB* tree, whereas nuclear-gene analyses placed it in association with *Chodsigoa* and *Soriculus* with weaker support. The results support the exclusion of *P. fumidus* from *Episoriculus*, but its exact position within Nectogalini should remain provisional pending broader sampling of *Soriculus*, *Pseudosoriculus*, *Chodsigoa*, and related taxa, together with nuclear markers.

At the tribal level, Soricini was recovered as a distinct lineage within Soricinae, but its relative position should be interpreted cautiously. Earlier mitochondrial studies recovered Soricini as a distinct tribe but left the branching order among major soricine tribes unresolved [43,44]. Later multilocus analyses generally supported the main soricine tribes, although the relative position of Soricini remained sensitive to analytical framework, as shown by its alternative placement in the ASTRAL tree of He et al. [4].

In the mitogenomic topology, Soricini was more consistent with the arrangement in which it is sister to the clade comprising Anourosoricini and Nectogalini (BS = 72%; PP = 1.00). However, because Notiosoricini was not included in the present analysis and the dataset consisted only of mitochondrial genomes, this result should be regarded as a mitochondrial phylogenetic hypothesis rather than a definitive resolution of tribal relationships within Soricinae. Within Soricini, phylogenetic relationships among *S. roboratus*, *S. daphaenodon*, *S. asper*, and *S. tundrensis* remained unresolved owing to the deficiency of mitogenome data for *S. asper*. The phylogenetic positions of the other three species were generally consistent with the topology reported by Wang Xuming, with *S. daphaenodon* and *S. tundrensis* forming a well-supported sister clade. For the clade consisting of *S. minutus*, *S. thibetanus*, *S. minutissimus*, and *S. gracillimus*, the results were inconsistent with findings from Wang Xuming’s previous studies, which were based on mtDNA, nDNA, and concatenated mitochondrial–nuclear datasets [48].

Previous phylogenetic studies have suggested that rapid diversification may have contributed to unresolved or weakly supported relationships within Nectogalini and Soricini [17,44,49,50,51]. Consistent with this, several nodes in these groups remain sensitive to marker choice, taxon sampling, and analytical framework. In the mitochondrial tree, *Chimarrogale* and *Nectogale* formed a strongly supported clade, whereas *N. fodiens* was not recovered as sister to this clade. Similarly, *P. fumidus* clustered with *S. nigrescens*, but its precise placement within Nectogalini remains uncertain in light of previous multilocus studies. These results highlight the usefulness of complete mitochondrial genomes for comparative analyses, while also indicating that broader nuclear genomic sampling will be needed to fully resolve these unstable relationships.

### 4.2. Lifestyle-Associated Mitogenomic Variation in Soricines

Semi-aquatic soricines showed higher AT content and T3s values, but lower C3s, G3s, and GC3s values than terrestrial soricines, and most of these differences remained significant after phylogenetic correction. Although A3s was also higher in semi-aquatic soricines, this difference did not remain significant after FDR correction. These patterns indicate that lifestyle-associated nucleotide-composition differences are particularly evident at third codon positions. This pattern is consistent with the concept of directional mutation pressure, in which relatively weakly constrained sites, especially synonymous third codon positions, are more likely to retain mutational signals than more constrained sites [40,52]. The stronger AT-rich signal in semi-aquatic soricines may also reflect differences in strand-specific nucleotide bias, as mitochondrial strand asymmetry has been linked to asymmetric mutation pressure during replication [53]. The neutrality analysis showed descriptively different regression slopes between semi-aquatic and terrestrial soricines. However, after accounting for phylogenetic relationships, the interaction between GC3s and lifestyle was not significant, indicating that the relationship between GC12 and GC3s did not differ significantly between the two ecological groups. Therefore, although semi-aquatic soricines exhibited a higher descriptive regression slope, the present results do not provide strong evidence that mutation pressure contributes proportionally more to codon usage in semi-aquatic lineages. Instead, the observed differences are more conservatively interpreted as lifestyle-associated variation in mitochondrial nucleotide composition and synonymous codon usage, the underlying evolutionary causes of which remain unresolved.

Terrestrial soricines exhibited higher CAI, Fop, and CBI values than semi-aquatic soricines, and these differences remained significant after phylogenetic correction, indicating a stronger bias toward preferred codon usage in terrestrial soricines. The Ka/Ks ratios of all 13 mitochondrial protein-coding genes were below 1, indicating pervasive purifying selection. However, phylogenetically corrected analyses revealed a gene-specific rather than uniform pattern of evolutionary-rate variation. *COX1* and *ND6* showed significantly higher Ka/Ks ratios in semi-aquatic soricines, whereas *ND3* and *ND4L* showed significantly lower ratios; no significant lifestyle-associated differences were detected for the remaining genes after FDR correction. These results therefore do not support a general relaxation of purifying selection in semi-aquatic soricines, but instead suggest that changes in selective constraint are heterogeneous among mitochondrial genes.

Such gene-specific variation may result from several non-mutually exclusive processes. Differences in demographic history can influence the efficacy of purifying selection because reductions in effective population size strengthen the effects of genetic drift and reduce the efficiency with which weakly deleterious variants are removed [54]. Therefore, demographic differences among lineages may contribute to variation in mitochondrial evolutionary rates independently of ecological lifestyle. In addition, ecological transitions may alter functional constraints on particular mitochondrial genes, potentially producing lineage- and gene-specific shifts in evolutionary rates rather than a uniform response across the mitogenome. Similar heterogeneous patterns of mitochondrial evolution have been reported in taxa occupying specialised ecological niches, including flightless birds [12], cave-dwelling fish [13], and semi-aquatic otters [55]. However, demographic history and changes in gene-specific functional constraint were not directly tested in the present study and should therefore be regarded as possible explanations rather than demonstrated mechanisms.

An additional consideration in interpreting mitochondrial compositional and codon-use variation is the structural heterogeneity of mitochondrial proteins. The 13 mitochondrial protein-coding genes contain distinct structural regions, including transmembrane helices and extramembrane domains, which may experience different evolutionary constraints. For example, thymine enrichment at the second codon position of transmembrane regions has been associated with hydrophobic amino-acid requirements for membrane embedding [56]. Therefore, whole-gene analyses may combine signals from regions with different structural constraints. Structural-domain effects were not separated in the present study, and their contribution to the observed nucleotide composition and codon-use patterns remains unclear. Future analyses incorporating domain-specific partitioning, such as TRAMPO-based approaches, together with broader taxon sampling and phylogenetically controlled methods, may help clarify the relative roles of structural constraints and ecological factors in mitochondrial genome evolution.

## 5. Conclusions

This study provides the complete mitogenome of *C*. *styani*, which is 17,207 bp in length and contains the typical vertebrate mitochondrial gene set. Phylogenetic analyses based on 13 protein-coding genes and two rRNA genes supported the monophyly of Soricinae and recovered *C. styani* as sister to *C. leander*, with *Chimarrogale* closely related to *Nectogale*. Comparative analyses of semi-aquatic and terrestrial soricines revealed lifestyle-associated differences in nucleotide composition, synonymous codon usage, amino-acid properties, and evolutionary rates. After phylogenetic correction, semi-aquatic soricines had higher AT content and T3s values, whereas terrestrial soricines showed a stronger bias toward preferred synonymous codons. Semi-aquatic soricines also showed higher average protein hydrophobicity. Evolutionary-rate differences were gene-specific rather than uniformly elevated in semi-aquatic soricines: *COX1* and *ND6* showed higher Ka/Ks ratios, whereas *ND3* and *ND4L* showed lower ratios. All 13 mitochondrial protein-coding genes had Ka/Ks ratios below 1, consistent with pervasive purifying selection. The present study has several limitations. The comparative dataset included only 11 species, and lifestyle categories remain strongly associated with phylogenetic lineage despite the use of PGLS. In addition, the analyses were based exclusively on mitochondrial genomes and did not separate potential structural-domain effects within mitochondrial proteins. Therefore, the observed differences should be interpreted as lifestyle-associated rather than strictly lifestyle-driven. Broader taxon sampling incorporating additional independent ecological transitions, nuclear genomic data, and structural-domain-specific analyses will be needed to further evaluate the relationship between ecological lifestyle and mitochondrial genome evolution in Soricinae.

## Figures and Tables

**Table 1:** General information on semi-aquatic and terrestrial soricines and their mitogenomes.

Tribe	Genera	Species	Acc. No.	Lifestyle
Nectogalini	*Chimarrogale*	*C. styani*	PX068317	Semi-aquatic
		*C. leander*	OP132391	Semi-aquatic
	*Nectogale*	*N. elegans*	NC023351	Semi-aquatic
	*Neomys*	*N. fodiens*	NC025559	Semi-aquatic
Soricini	*Sorex*	*S. araneus*	NC027963	Terrestrial
		*S. caecutiens*	MF374796	Terrestrial
		*S. cylindricauda*	KF696672	Terrestrial
		*S. gracillimus*	NC037859	Terrestrial
		*S*. *minutus*	MN122904	Terrestrial
		*S*. *tundrensis*	NC025327	Terrestrial
		*S*. *alpinus*	NC087795	Terrestrial

## Data Availability

The complete mitogenome of *C. styani* is available in GenBank (accession number PX068317), and the voucher specimen HP2405007 is deposited in the Institute of Pathogens and Vectors, Dali University, China.

## Supplementary Materials

The following supporting information can be downloaded at https://www.mdpi.com/article/10.3390/genes17101244/s1. Figure S1: Nucleotide substitution saturation analysis of the PCG + rRNA dataset; Figure S2: ENC-plots of four semi-aquatic shrew species: (a) *C. styani*; (b) *Chimarrogale leander*; (c) *Nectogale elegans*; (d) *N. fodiens*; Figure S3: ENC-plots of seven terrestrial shrew species. (a) *Sorex araneus*; (b) *Sorex cylindricauda*; (c) *Sorex gracillimus*; (d) *Sorex caecutiens*; (e) *Sorex alpinus*; (f) *Sorex minutus*; (g) *Sorex tundrensis*; Figure S4: PR2-plots of four semi-aquatic shrew species. (a) *C. styani*; (b) *C. leander*; (c) *N. elegans*; (d) *N. fodiens*; Figure S5: PR2-plots of seven terrestrial shrew species. (a) *S. araneus*; (b) *S. cylindricauda*; (c) *S. gracillimus*; (d) *S. caecutiens*; (e) *S. alpinus*; (f) *S. minutus*; (g) *S. tundrensis*; Figure S6: Sequencing depth and coverage profile of the assembled *C. styani* mitochondrial genome; Table S1: Geographic coordinates of sampling localities from this study and occurrence records compiled from the literature, museum collections, and online databases [57,58,59,60,61,62,63,64,65]; Table S2: Morphological comparison between semi-aquatic and terrestrial shrews; Table S3: Mitogenomic data used in the phylogenetic analyses of Soricidae. Lifestyle assignments were compiled from books and published literature; blank cells indicate taxa for which lifestyle information was unavailable; Table S4: Phylogenetic generalized least-squares (PGLS) analyses of mitochondrial genomic, nucleotide-composition, codon-usage, and protein-property traits; Table S5: Gene-specific PGLS analyses of Ka/Ks ratios for the 13 mitochondrial protein-coding genes; Table S6: Phylogenetically controlled models for the neutrality and ENC–GC3s relationships; Table S7: Exploratory PGLS analyses of RSCU summary counts.
