# Supplementary material for: Complete Mitogenome of Chimarrogale styani, Phylogenetic Placement Within Soricinae, and Exploratory Mitogenomic Comparisons
Source: Genes (Basel). 2026 Oct 9;17(10):1244. doi: 10.3390/genes17101244 (PMC13704500; doi:10.3390/genes17101244)
Supplement: Supplementary file 1 [file genes-17-01244-s001.zip › genes-4603063-supplementary.pdf]

Supplementary Materials

Complete Mitogenome of *Chimarrogale styani*, Phylogenetic Placement within Soricinae, and Exploratory Mitogenomic Comparisons

Xiujia Wu, Tao Wen, Guangzhi Chen and Wenge Dong *

Key Laboratory of Ectoparasite Systematics and Evolution of Yunnan Provincial Education Department, Institute of Pathogens and Vectors, Dali University, Dali 671000, China

* Correspondence: dongwenge2740@sina.com

This file contains Figures S1–S6 and Tables S1–S7.

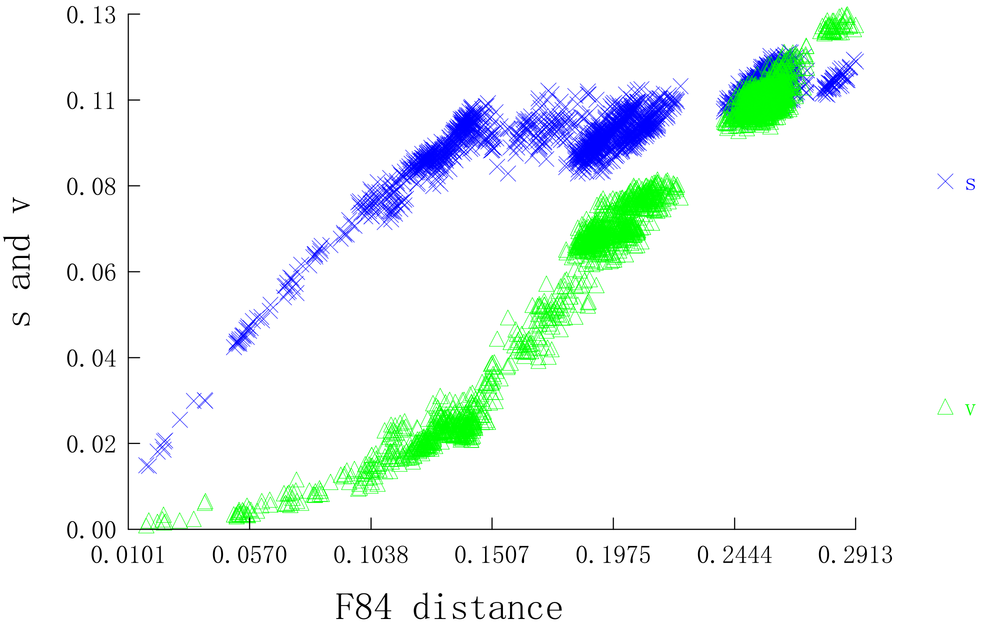

Figure S1. Nucleotide substitution saturation analysis of the PCG + rRNA dataset.

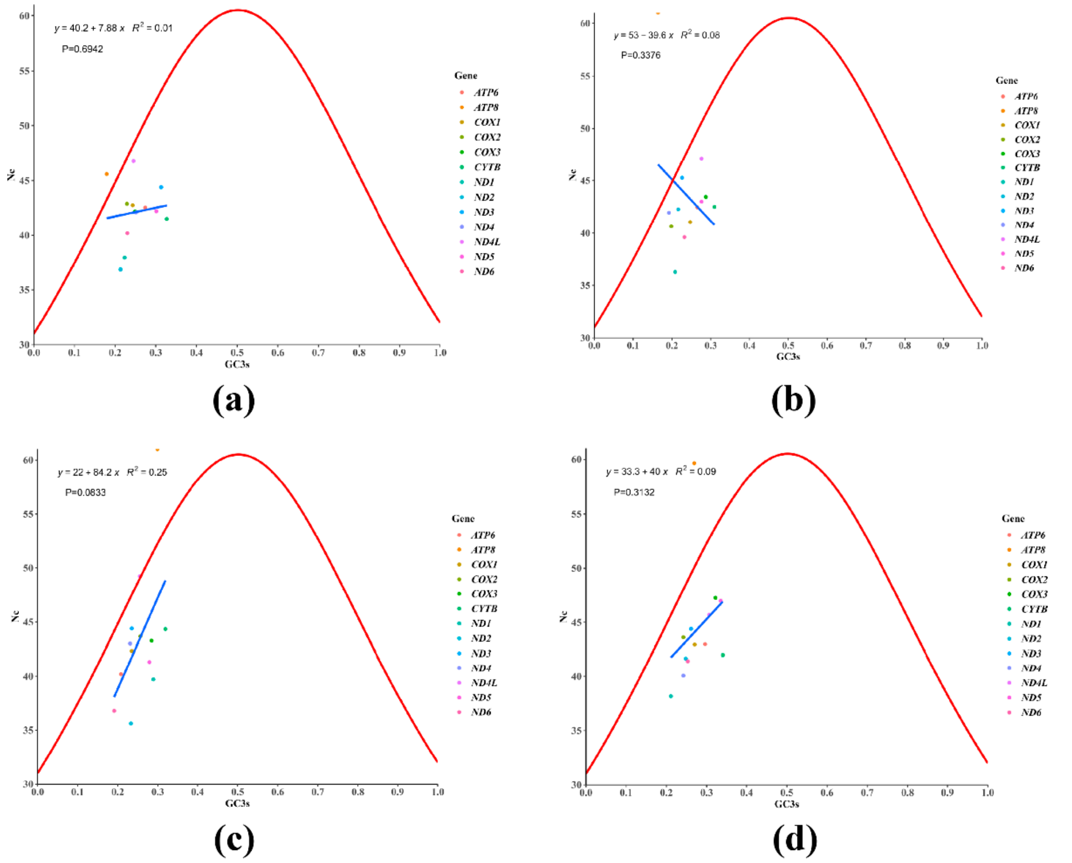

Figure S2. ENC-plots of four semi-aquatic shrew species: (a) *C. styani*; (b) *Chimarrogale leander*; (c) *Nectogale elegans*; (d) *N. fodiens*.

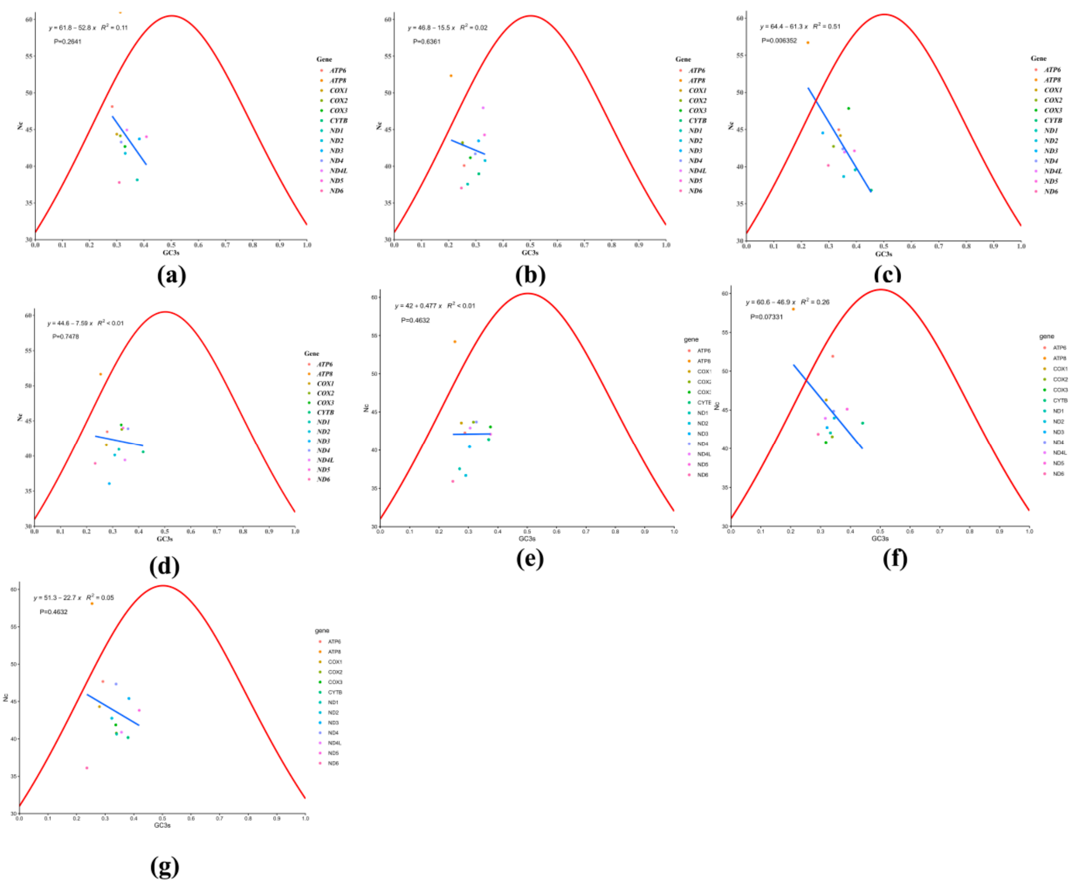

Figure S3. ENC-plots of seven terrestrial shrew species. (a) *Sorex araneus*; (b) *Sorex cylindricauda*; (c) *Sorex gracillimus*; (d) *Sorex caecutiens*; (e) *Sorex alpinus*; (f) *Sorex minutus*; (g) *Sorex tundrensis*.

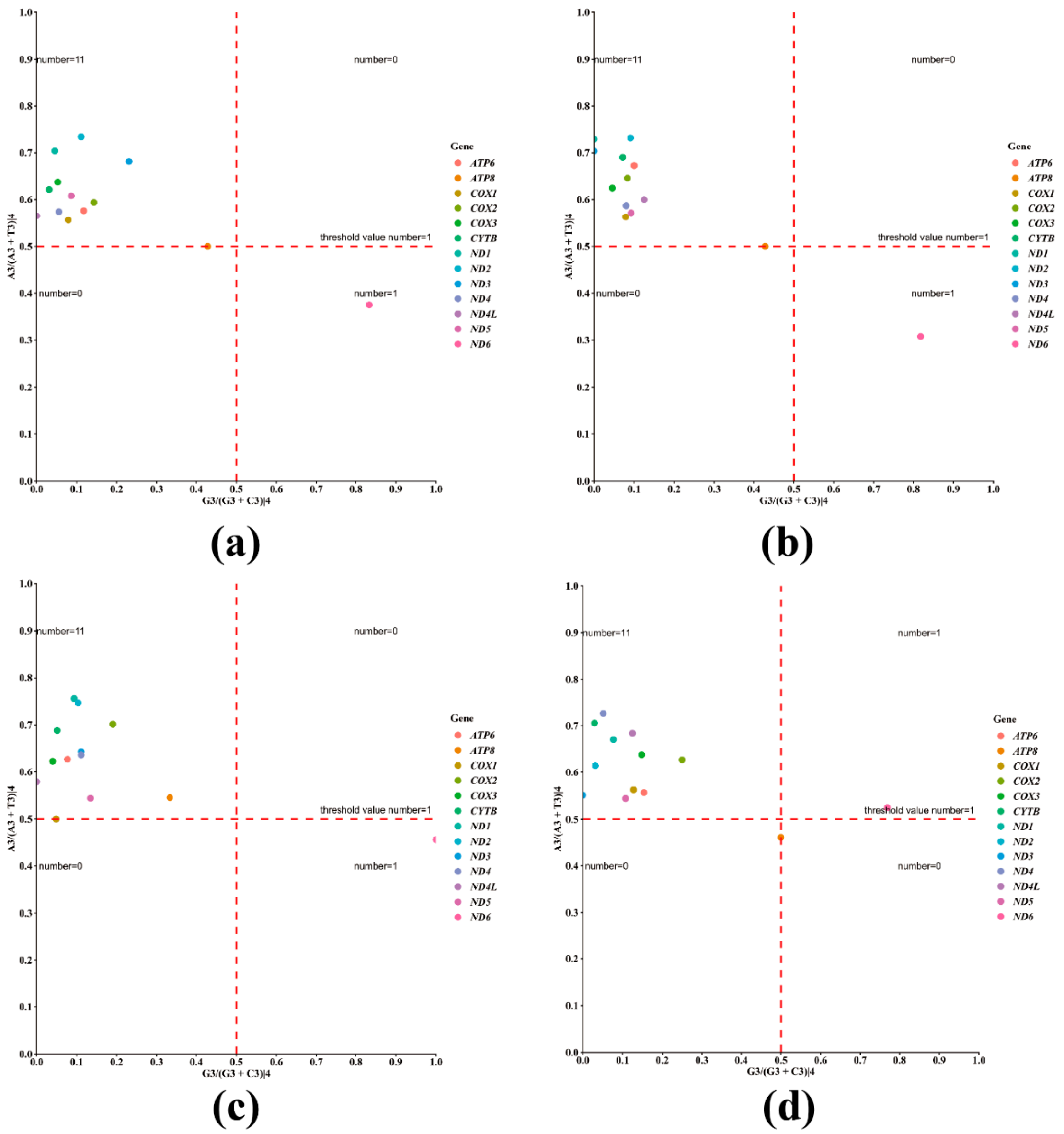

Figure S4. PR2-plots of four semi-aquatic shrew species. (a) *C. styani*; (b) *C. leander*; (c) *N. elegans*; (d) *N. fodiens*.

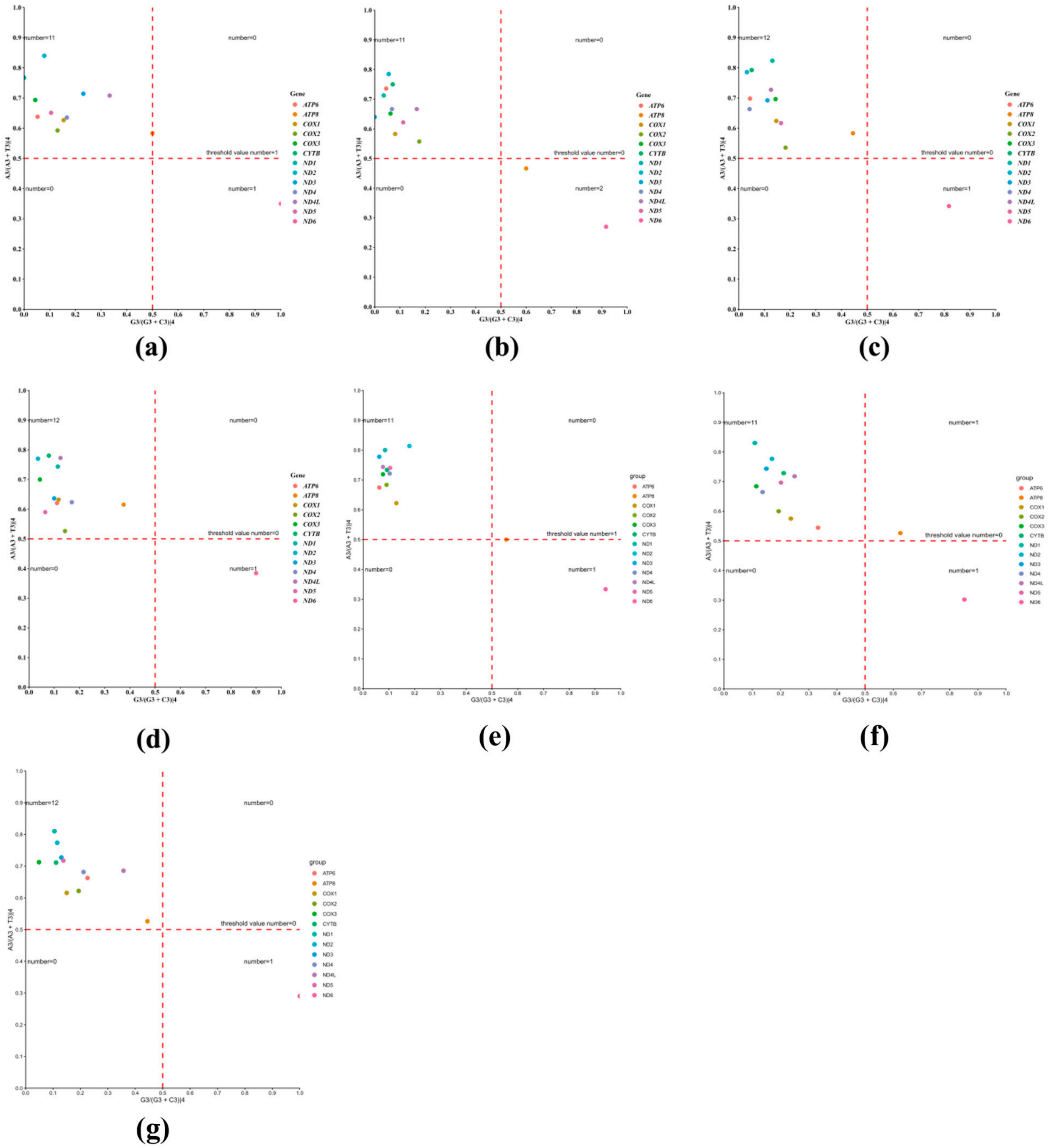

Figure S5. PR2-plots of seven terrestrial shrew species. (a) *S. araneus*; (b) *S. cylindricauda*; (c) *S. gracillimus*; (d) *S. caecutiens*; (e) *S. alpinus*; (f) *S. minutus*; (g) *S. tundrensis*.

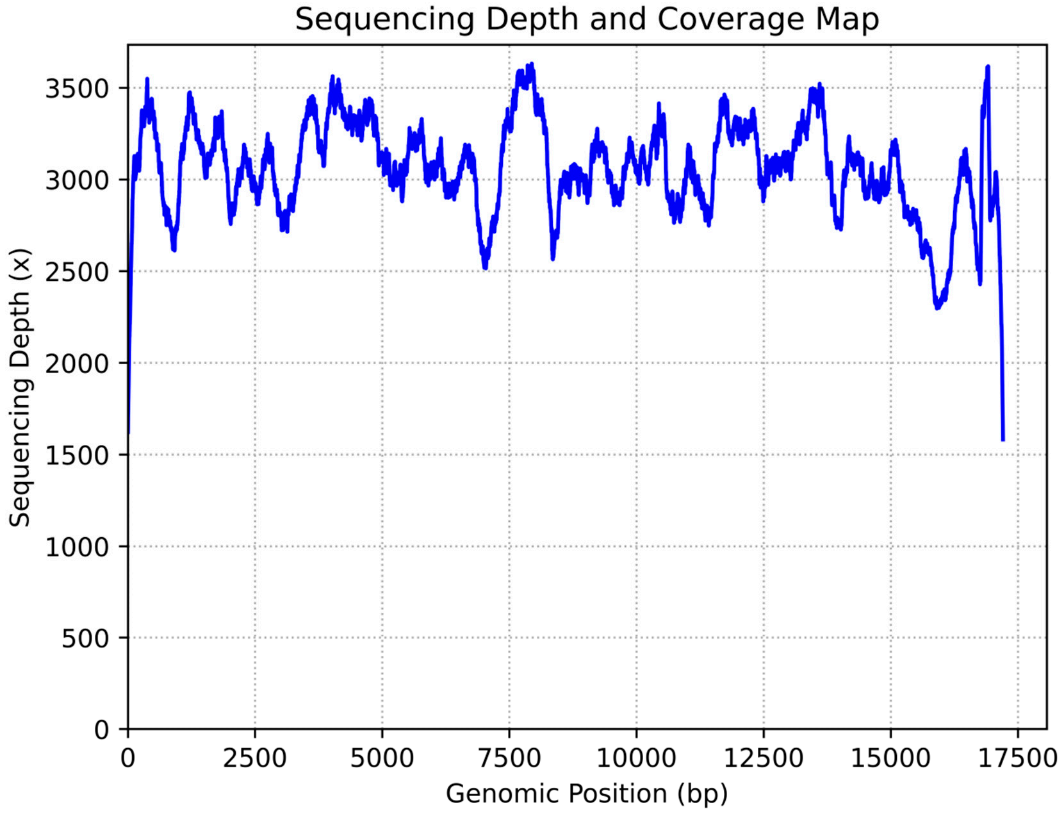

(1) Total genome length = 17,207 bp

(2) Average depth = 3063.31 x

(3) Maximal depth = 3632 x

(4) Minimal depth = 1582 x

Figure S6. Sequencing depth and coverage profile of the assembled *C. styani* mitochondrial genome.

Table S1. Geographic coordinates of sampling localities from this study and occurrence records compiled from the literature, museum collections, and online databases.

No.	Record category	Latitude	Longitude	Locality	Source
1	this study	28.280577	98.888892	Hongpo Village, Deqin County, Diqing Tibetan Autonomous Prefecture, Yunnan Province, China	PX068317
2	published genetic samples (BRCA1)	26.358	99.381	Lanping, Yunnan, China	ON219792
3	published genetic samples (BRCA1)	26.358	99.381	Lanping, Yunnan, China	ON219791
4	preserved specimen	32.42	104.52	Yang-liu-pa, Sichuan Province, China,	BMNH 99.3.1.8 (holotype)
5	online databases	26.06	26.06	Pianma, Lushui, Yunnan, China	Scientific Data Center, Kunming Institute of Zoology, CAS
6	online databases	24.05	101.48	Ailao Mountain Nature Reserve, Yunnan, China	Scientific Data Center, Kunming Institute of Zoology, CAS
7	online databases	26.98	98.85	Nujiang Nature Reserve, Yunnan, China	Scientific Data Center, Kunming Institute of Zoology, CAS
8	preserved specimen	27.74	98.5	Burma, Kachin Dist., road to Chimeli Pass,1(AMNH 114830)	(S.Hoffmann 1987)
9	preserved specimen	26.15	98.6	Burma, Kachin Dist, Gangfang(AMNH 114827)	(S.Hoffmann 1987)
10	preserved specimen	26.16076	98.46256	Burma, Kachin Dist, Gangfang(AMNH 114828)	(S.Hoffmann 1987)
11	preserved specimen	26.16076	98.46256	Burma, Kachin Dist, Imaw Bum (AMNH 114829)	(S.Hoffmann 1987)
12	preserved specimen	30.983	101.133	China, Tao Fu (= Dawu), (AMNH 113531)	(S.Hoffmann 1987)
13	online databases	26.5	98.5	Wulaw Pass, Naung Chang Valley, West Burma, currently Myanmar	GBIF
14	online databases	26.5	98.5	Wulaw Pass, Naung Chang Valley, West Burma, currently Myanmar	GBIF
15	literature records	29.08	107.27	Jinfo Mountain Nature Reserve, Sichuan, China	(Peng et al. 1996)
16	literature records	33.08	104.32	Wujiao Nature Reserve, Sichuan, China	(JIANG, LI, and HU 2013)
17	literature records	33.17	103.92	Jiuzhaigou Nature Reserve, Sichuan, China	(LIU et al. 2005)
18	literature records	32.58	32.58	Tangjiahe Nature Reserve, Sichuan, China	(Hu 1999)
19	literature records	29.57	106.55	Chongqing Municipality, China	(Chongqing Municipal Forestry Bureau and Chongqing Municipal Agricultural and Rural Affairs Commission 2023)
20	literature records	29.22	94.22	Milin County, Tibet Autonomous Region, China	(HE et al. 2017)

Table S2. Morphological comparison between semi-aquatic and terrestrial shrews.

Character	Semi-aquatic				Terrestrial						
	Chimarrogale styani	*Chimarrogale leander*	*Nectogale elegans*	*Neomys fodiens*	*Sorex cylindricauda*	*Sorex caecutiens*	*Sorex gracillimus*	*Sorex araneus*	*Sorex minutus*	*Sorex alpinus*	*Sorex tundrensis*
Head-body length	80-105mm	105mm	88-115mm	75-105mm	52-84mm	50-73mm	45-66mm	56-82mm	40-64mm	62-77mm	61-86mm
Body weight	17-26.9g	22g	Not described	8.5-25g	Not described	4-12.5g	2.5-5.3g	5-14.5g	2.6-7g	5.5-11.5g	8-11.5g
Tail length	91-100mm	86mm	100-104mm	58-73mm	45 ~62 mm	28-45 mm	33-45 mm	37-52 mm	33-45mm	60-75mm	20-38mm
Hind foot length	20-22 mm	22 mm	25-27 mm	16-21 mm	12.5-16 mm	10-12 mm	9-12 mm	12-14 mm	9-12mm	12-17mm	13-15mm
Ear length	5-6 mm	Not described	Absence of the auricle	Not described	6 mm	Not described	Not described	Not described	Not described	Not described	Not described
Ear valve	Not described	Yes	Yes	Not described	No	No	No	No	No	No	No
Dental pigmentation	No	No	No	Red	Red	Red	Red	Red	Red	Red	Red
Webbed hindfeet	Yes	Yes	Yes	Yes	No	No	No	No	No	No	No
Stiff hairs	a fringe of stiff hairs on the lateral edges of their forefeet and hindfeet and each toe	a fringe of stiff hairs on the lateral edges of their forefeet and hindfeet and each toe	a fringe of stiff hairs on the lateral edges of their forefeet and hindfeet and each toe	a fringe of stiff hairs on the lateral edges of their forefeet and hindfeet and each toe	No	No	No	No	No	No	No
Dental formula	3.1.1.3/2.0.1.3=28	3.1.1.3/2.0.1.3=28	3.1.1.3/2.0.1.3=28	3.1.2.3/2.0.1.3=30	3.1.3.3/2.0.1.3=32	3.1.3.3/2.0.1.3=32	3.1.3.3/2.0.1.3=32	3.1.3.3/2.0.1.3=32	3.1.3.3/2.0.1.3=32	3.1.3.3/2.0.1.3=32	3.1.3.3/2.0.1.3=32
Awn hairs	The dorsal side is scattered with white awn hairs, particularly conspicuous on the rump.	The dorsal side is scattered with white awn hairs, particularly conspicuous on the rump.	The dorsal side is scattered with white awn hairs, particularly conspicuous on the rump.	The dorsal side is scattered with white awn hairs, particularly conspicuous on the rump.	No	No	No	No	No	No	No

Note: "Not described" indicates that the character was not reported in the sources consulted.

Table S3. Mitogenomic data used in the phylogenetic analyses of Soricidae. Lifestyle assignments were compiled from books and published literature; blank cells indicate taxa for which lifestyle information was unavailable.

Tribe	Genus	Species	Accession No.	Lifestyle
Anourosoricini	Anourosorex	*Anourosorex squamipes*	NC_024563	semi-fossorial
Blarinini	Blarina	*Blarina brevicauda*	NC_042734	
Blarinini	Blarina	*Blarina hylophaga*	NC_042694	
Blarinellini	Blarinella	*Blarinella quadraticauda*	NC_023950	semi-fossorial
Blarinellini	Blarinella	*Blarinella wardi*	NC_041145	semi-fossorial
Nectogalini	Chimarrogale	*Chimarrogale leander*	OP132391	semi-aquatic
Nectogalini	Chimarrogale	*Chimarrogale styani*	PX068317	semi-aquatic
Nectogalini	Chodsigoa	*Chodsigoa hoffmanni*	MK940327	
Nectogalini	Chodsigoa	*Chodsigoa hypsibia*	NC_060870	
Nectogalini	Chodsigoa	*Chodsigoa parva*	NC_053858	
Nectogalini	Chodsigoa	*Chodsigoa smithii*	MN038168	semi-arboreal
Crocidurinae	Crocidura	*Crocidura anhuiensis*	NC_088563	
Crocidurinae	Crocidura	*Crocidura armenica*	OR449074	
Crocidurinae	Crocidura	*Crocidura attenuata*	NC_026204	terrestrial
Crocidurinae	Crocidura	*Crocidura baileyi*	PQ863111	
Crocidurinae	Crocidura	*Crocidura beata*	NC_027249	
Crocidurinae	Crocidura	*Crocidura dongyangjiangensis*	NC_056167	
Crocidurinae	Crocidura	*Crocidura fuliginosa*	NC_042762	
Crocidurinae	Crocidura	*Crocidura glassi*	PQ863104	
Crocidurinae	Crocidura	*Crocidura grayi*	NC_027247	
Crocidurinae	Crocidura	*Crocidura gueldenstaedtii*	ON682395	
Crocidurinae	Crocidura	*Crocidura harenna*	PQ863112	
Crocidurinae	Crocidura	*Crocidura lasiura*	NC_029329	semi-aquatic
Crocidurinae	Crocidura	*Crocidura leucodon*	ON682410	
Crocidurinae	Crocidura	*Crocidura lucina*	PQ863110	
Crocidurinae	Crocidura	*Crocidura macmillani*	PQ863113	
Crocidurinae	Crocidura	*Crocidura mindorus*	NC_027248	
Crocidurinae	Crocidura	*Crocidura negrina*	NC_027245	
Crocidurinae	Crocidura	*Crocidura ninoyi*	NC_027244	
Crocidurinae	Crocidura	*Crocidura orientalis*	NC_027242	
Crocidurinae	Crocidura	*Crocidura palawanensis*	NC_027243	
Crocidurinae	Crocidura	*Crocidura panayensis*	NC_027246	
Crocidurinae	Crocidura	*Crocidura shantungensis*	NC_021398	
Crocidurinae	Crocidura	*Crocidura sibirica*	MH349094	
Crocidurinae	Crocidura	*Crocidura suaveolens mimula*	ON682427	
Crocidurinae	Crocidura	*Crocidura tanakae*	NC_046831	terrestrial
Crocidurinae	Crocidura	*Crocidura wuchihensis*	NC_079638	terrestrial
Blarinini	Cryptotis	*Cryptotis goldmani*	MZ457411	
Blarinini	Cryptotis	*Cryptotis gracilis*	MZ457413	
Blarinini	Cryptotis	*Cryptotis lacertosus*	MZ457415	
Blarinini	Cryptotis	*Cryptotis mayensis*	MZ457409	
Blarinini	Cryptotis	*Cryptotis merriami*	MZ457417	
Blarinini	Cryptotis	*Cryptotis mexicanus*	MZ457410	

Tribe	Genus	Species	Accession No.	Lifestyle
Blarinini	Cryptotis	*Cryptotis nigrescens*	MZ457412	
Blarinini	Cryptotis	*Cryptotis oreoryctes*	MZ457416	
Blarinini	Cryptotis	*Cryptotis parvus*	MZ457414	
Blarinini	Cryptotis	*Cryptotis tropicalis*	MZ457418	
Nectogalini	Episoriculus	*Episoriculus caudatus*	NC_026131	semi-arboreal
Nectogalini	Episoriculus	*Episoriculus leucops*	NC_056333	
Nectogalini	Episoriculus	*Episoriculus macrurus*	NC_029840	semi-arboreal
Nectogalini	Episoriculus	*Episoriculus umbrinus*	NC_073538	
Nectogalini	Nectogale	*Nectogale elegans*	NC_023351	semi-aquatic
Nectogalini	Neomys	*Neomys fodiens*	NC_025559	semi-aquatic
Blarinellini	Parablarinella	*Parablarinella griselda*	NC_042749	
Nectogalini	Pseudosoriculus	*Pseudosoriculus fumidus*	NC_003040	semi-arboreal
Soricini	Sorex	*Sorex alpinus*	NC_087795	terrestrial
Soricini	Sorex	*Sorex caecutiens*	MF374796	terrestrial
Soricini	Sorex	*Sorex cylindricauda*	KF696672	terrestrial
Soricini	Sorex	*Sorex daphaenodon*	NC_044107	
Soricini	Sorex	*Sorex excelsus*	KY676803	
Soricini	Sorex	*Sorex gracillimus*	NC_037859	terrestrial
Soricini	Sorex	*Sorex isodon*	MG983792	
Soricini	Sorex	*Sorex minutissimus*	NC_042196	
Soricini	Sorex	*Sorex roboratus*	NC_034808	
Soricini	Sorex	*Sorex sinalis*	NC_037174	
Soricini	Sorex	*Sorex thibetanus*	NC_064993	
Soricini	Sorex	*Sorex tundrensis*	NC_025327	terrestrial
Soricini	Sorex	*Sorex unguiculatus*	NC_005435	
Nectogalini	Soriculus	*Soriculus nigrescens*	NC_052688	semi-fossorial
Soricini	Sorex	*Sorex araneus*	NC_027963	terrestrial
Soricini	Sorex	*Sorex minutus*	MN122904	terrestrial
Crocidurinae	Suncus	*Suncus murinus*	NC_024604	
outgroup	Uropsilus	*Uropsilus gracilis*	NC_031330	
outgroup	Uropsilus	*Uropsilus investigator*	NC_060485	

Table S4. Phylogenetic generalized least-squares (PGLS) analyses of mitochondrial genomic, nucleotide-composition, codon-usage, and protein-property traits.

Trait	n	Estimate	SE	df	t	P	BH-FDR q	Pagel's λ
GC12	11	-0.0117	0.0016	9	-7.401	<0.0001	0.0006	0.00
GC1s	11	-0.0212	0.0030	9	-7.006	<0.0001	0.0006	0.00
T3s	11	0.0678	0.0102	9	6.653	<0.0001	0.0006	0.00
AT-skew	11	-0.0299	0.0049	9	-6.111	0.0002	0.0007	0.00
PR2 A/(A+T)	11	-0.0384	0.0063	9	-6.088	0.0002	0.0007	0.00
C3s	11	-0.0660	0.0111	9	-5.919	0.0002	0.0007	0.00
AT content (%)	11	2.4536	0.4472	9	5.486	0.0004	0.0010	0.00
GC3s	11	-0.0716	0.0132	9	-5.433	0.0004	0.0010	0.00
CAI	11	-0.0107	0.0023	9	-4.644	0.0012	0.0026	0.00
Fop	11	-0.0332	0.0072	9	-4.604	0.0013	0.0026	0.00
CBI	11	-0.0556	0.0125	9	-4.467	0.0016	0.0028	0.00
GC-skew	11	0.0196	0.0047	9	4.188	0.0023	0.0039	0.00
GRAVY	11	0.0127	0.0033	9	3.866	0.0038	0.0059	0.00
G3s	11	-0.0214	0.0070	9	-3.078	0.0132	0.0187	0.00
GC2s	11	-0.0022	0.0007	9	-3.038	0.0141	0.0187	0.00
A3s	11	0.0212	0.0093	9	2.281	0.0485	0.0606	0.00
ENC	11	-1.0561	0.4915	9	-2.149	0.0602	0.0708	0.00
Aromaticity	11	0.0008	0.0005	9	1.749	0.1143	0.1270	0.00
PR2 G/(G+C)	11	-0.0151	0.0116	9	-1.304	0.2247	0.2366	0.00
Mitogenome size (bp)	11	298.29	434.43	9	0.687	0.5096	0.5096	0.00

Note: Terrestrial soricines were the reference group. Positive estimates indicate higher values in semi-aquatic soricines; negative estimates indicate lower values. P values were adjusted within this family of tests using the Benjamini–Hochberg false-discovery-rate procedure. Pagel's λ was selected by maximum-likelihood profile search from 0 to 1 in increments of 0.01.

Table S5. Gene-specific PGLS analyses of Ka/Ks ratios for the 13 mitochondrial protein-coding genes.

Gene	n	Estimate	SE	df	t	P	BH-FDR q	Direction	Pagel's λ
COX1	11	0.00138	0.00025	9	5.526	0.0004	0.0044	Higher in semi-aquatic	0.00
ND3	11	-0.04326	0.00878	9	-4.930	0.0008	0.0044	Lower in semi-aquatic	0.00
ND6	11	0.03830	0.00804	9	4.763	0.0010	0.0044	Higher in semi-aquatic	0.00
ND4L	11	-0.02709	0.00783	9	-3.462	0.0071	0.0232	Lower in semi-aquatic	0.00
COX3	11	-0.00286	0.00128	9	-2.241	0.0518	0.1346	Lower in semi-aquatic	0.00
COX2	11	-0.00035	0.00024	9	-1.460	0.1784	0.3865	Lower in semi-aquatic	0.00
ATP8	11	0.05855	0.04802	9	1.219	0.2537	0.4712	Higher in semi-aquatic	0.00
ND1	11	0.00176	0.00196	9	0.894	0.3944	0.5977	Higher in semi-aquatic	1.00
ATP6	11	0.00798	0.00931	9	0.857	0.4138	0.5977	Higher in semi-aquatic	1.00
ND4	11	0.00033	0.00050	9	0.661	0.5249	0.6824	Higher in semi-aquatic	0.00
CYTB	11	-0.00143	0.00356	9	-0.401	0.6981	0.7938	Lower in semi-aquatic	0.00
ND2	11	-0.00434	0.01231	9	-0.352	0.7328	0.7938	Lower in semi-aquatic	0.00
ND5	11	0.00186	0.00904	9	0.206	0.8412	0.8412	Higher in semi-aquatic	0.00

Note: Terrestrial soricines were the reference group. P values were adjusted across the 13 gene-specific models using the Benjamini–Hochberg procedure.

Table S6. Phylogenetically controlled models for the neutrality and ENC–GC3s relationships.

Model	Term	Estimate	SE	df	t	P	Pagel's λ
Neutrality model (GC12 response)	(Intercept)	0.3902	0.0095	7	41.171	<0.0001	0.00
Neutrality model (GC12 response)	GC3s	0.0722	0.0285	7	2.532	0.0391	0.00
Neutrality model (GC12 response)	Lifestyle: semi-aquatic	-0.0342	0.0191	7	-1.792	0.1162	0.00
Neutrality model (GC12 response)	Interaction between GC3s and lifestyle	0.1063	0.0697	7	1.526	0.1709	0.00
ENC–GC3s model (ENC response)	(Intercept)	36.4656	3.4101	7	10.693	<0.0001	0.00
ENC–GC3s model (ENC response)	GC3s	22.2967	10.2626	7	2.173	0.0664	0.00
ENC–GC3s model (ENC response)	Lifestyle: semi-aquatic	-5.7926	6.8628	7	-0.844	0.4265	0.00
ENC–GC3s model (ENC response)	Interaction between GC3s and lifestyle	24.3549	25.0743	7	0.971	0.3638	0.00

Note: The neutrality model included GC3s, lifestyle, and their interaction as predictors of GC12. The ENC–GC3s model included GC3s, lifestyle, and their interaction as predictors of ENC.

Table S7. Exploratory PGLS analyses of RSCU summary counts.

Trait	n	Estimate	SE	df	t	P	BH-FDR q	Pagel's λ
Number of preferred codons (RSCU > 1)	11	0.8929	0.5762	9	1.549	0.1557	0.1557	0.00
Number of strongly overrepresented codons (RSCU > 1.6)	11	1.5714	0.6512	9	2.413	0.0390	0.0781	0.00

Note: These analyses are exploratory because the response variables are counts. Neither comparison remained significant after FDR correction.
